# Coaxial Direct Ink Writing of Cholesteric Liquid Crystal Elastomers in 3D Architectures

**DOI:** 10.1002/adma.202416621

**Published:** 2025-01-26

**Authors:** Alicia Ng, Rodrigo Telles, Katherine S. Riley, Jennifer A. Lewis, Caitlyn C. Cook, Elaine Lee, Shu Yang

**Affiliations:** ^1^ Department of Materials Science and Engineering University of Pennsylvania 3231 Walnut Street Philadelphia PA 19104 USA; ^2^ John A. Paulson School of Engineering and Applied Sciences and Wyss Institute for Biologically Inspired Engineering Harvard University Cambridge MA 02138 USA; ^3^ Lawrence Livermore National Laboratory Livermore CA 94550 USA

**Keywords:** 3D architectures, cholesteric liquid crystal elastomers, core‐shell, direct ink writing, mechanochromic, passive sensing

## Abstract

Cholesteric liquid crystal elastomers (CLCEs) hold great promise for mechanochromic applications in anti‐counterfeiting, smart textiles, and soft robotics, thanks to the structural color and elasticity. While CLCEs are printed via direct ink writing (DIW) to fabricate free‐standing films, complex 3D structures are not fabricated due to the opposing rheological properties necessary for cholesteric alignment and multilayer stacking. Here, 3D CLCE structures are realized by utilizing coaxial DIW to print a CLC ink within a silicone ink. By tailoring the ink compositions, and thus, the rheological properties, the cholesteric phase rapidly forms without an annealing step, while the silicone shell provides encapsulation and support to the CLCE core, allowing for layer‐by‐layer printing of self‐supported 3D structures. As a demonstration, free‐standing bistable thin‐shell domes are printed. Color changes due to compressive and tensile stresses can be witnessed from the top and bottom of the inverted domes, respectively. When the domes are arranged in an array and inverted, they can snap back to their base state by uniaxial stretching, thereby functioning as mechanical sensors with memory. The additive manufacturing platform enables the rapid fabrication of 3D mechanochromic sensors thereby expanding the realm of potential applications for CLCEs.

## Graphical abstract

A low‐viscosity cholesteric liquid crystal (CLC) ink and a silicone ink are prepared for coaxial direct ink writing of 3D strain sensors. The silicone shell supports the CLC core to enable layer‐by‐layer printing in addition to enhancing the mechanical properties of the core. Monostable and bistable thin‐shell domes are printed to demonstrate the 3D printing capabilities and mechanochromic behaviors.

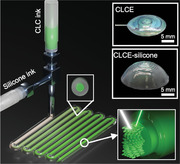

## 1 Introduction

Mechanochromic strain sensors are being widely developed in the form of anti‐counterfeiting devices,^[^ ^1^, ^2^ ^]^ smart textiles,^[^ ^3^ ^]^ health monitoring devices,^[^ ^4^ ^]^ and soft robotics^[^ ^5^, ^6^ ^]^ to improve quality of life. Color changes provide visual cues that are easily detected by the human eye. Colors in mechanochromic sensors can be chemical^[^ ^7^ ^]^ or structural‐based.^[^ ^8^, ^9^, ^10^, ^11^, ^12^ ^]^ Compared to chemical‐based chromogenic materials, such as pigments and dyes, structural colors have longer lifetimes and are more environmentally friendly. Cholesteric liquid crystal elastomers (CLCEs) are unique structural‐based chromogenic materials. CLCEs are produced by lightly crosslinking reactive cholesteric liquid crystal (CLC) monomers and oligomers. Due to their anisotropy and elasticity, CLCEs can passively and reversibly respond in real time to mechanical stress, temperature, or other external stimuli. Their color‐changing behavior is of interest for potential applications in cryptography,^[^ ^1^, ^5^ ^]^ smart wearables,^[^ ^3^, ^13^ ^]^ and soft robotics.^[^ ^14^, ^15^ ^]^ The cholesteric (also known as chiral nematic) phase of CLCEs is produced by the spontaneous self‐assembly of LC mesogens and chiral dopants into a periodic helical structure. The reflected wavelength of light, λ, is directly proportional to the average refractive index, 〈*n*〉, and the helical pitch, *P*, as defined by the Bragg equation:

(1) $$\lambda =\langle n\rangle P\cos\theta$$

where *θ* is the angle of incidence between the light source and helical axis. The pitch is inversely proportional to the concentration of chiral dopant, *c*, as shown in the equation for helical twisting power, $HTP=\frac{1}{P\times c}\;$. Hence, CLCEs can be tuned to reflect the light by simply changing *c*. Mechanical stress can distort the helix and alter the pitch, therefore producing a passive color change governed by Equation 1. For example, tension applied perpendicular to the helical axis causes a blue shift, due to the Poisson's effect causing compression along the helical axis; conversely, compression applied perpendicular to the helical axis causes a red shift. Compression applied parallel to the helical axis, however, directly compresses the pitch and causes a blue shift.

A monodomain cholesteric phase can be achieved in several ways. Most commonly, surface alignment^[^ ^5^, ^16^ ^]^ and anisotropic deswelling^[^ ^17^, ^18^ ^]^ are used to produce films (microns thin to millimeters thick), where the CLC phase is retained in a fully crosslinked 2D geometry, or lightly crosslinked in a film before mechanical manipulation into different shapes such as a spiral ribbon^[^ ^15^ ^]^ or a “beetle” shape^[^ ^19^ ^]^ and then crosslinking fully. Additive manufacturing by direct ink writing (DIW)^[^ ^20^, ^21^ ^]^ shows the most promise for advancing CLCE fabrication due to simplicity, speed, and capacity for local control of alignment with good feature resolution. During printing, CLC inks are subjected to shear and extensional flows that provide local control over the extruded CLC phase, thus enabling the generation of intricate designs^[^ ^22^, ^23^ ^]^ that would otherwise be difficult to produce. Despite significant efforts in DIW of LCEs, only a handful studies have reported DIW of CLCEs.^[^ ^22^, ^23^, ^24^, ^25^, ^26^, ^27^, ^28^ ^]^ Sol et al. developed humidity‐responsive CLCE films^[^ ^24^ ^]^ and photochromic CLCE actuators with tilted cholesteric alignment^[^ ^25^ ^]^ using a heated syringe at 70 °C and a heated bed at 44 or 39 °C. The photo‐responsive films are only 30–65 µm thin, hence macroscopic actuation is possible. However, an annealing time of 15 min on the 44 °C bed is necessary for proper cholesteric alignment. Choi et al. utilized a low‐molecular‐weight chiral dopant to produce solvent‐free ink and print elastic mechanochromic films with a slanted helix.^[^ ^23^ ^]^ However, the ink's high viscosity necessitated a nozzle temperature of 75 °C and an annealing step of 10 min at 40 °C to allow for CLC alignment. Yang et al. developed mechanochromic sensors by printing CLCEs onto elastomeric films via evaporation‐assisted DIW.^[^ ^27^ ^]^ Although they printed 2D patterns from CLCE without using heat, a long duration (at least 10 min) is required for the solvent‐driven CLC alignment, making it challenging to print 3D structures layer‐by‐layer. Li et al. fabricated mechanoresponsive core‐shell CLCE, with a CLCE shell from solvent‐driven CLC alignment and a water‐soluble polymer core that can be removed to generate a hollow core with enhanced color contrast.^[^ ^28^ ^]^ Like other literature, the structures remain planar. Fabricating 3D CLCE structures remains difficult due to the orthogonal rheological property requirements for aligning CLCs and printing multiple layers. Oligomeric CLC inks developed to date have high viscosities due to their large mesogens and relatively long chains. Their high viscosities hinder mesogen rotation, which is necessary for their self‐assembly into the cholesteric phase. Hence, prior to UV curing to form an LCE network, an additional annealing step must be performed with heat, time,^[^ ^27^ ^]^ or both,^[^ ^22^, ^23^, ^24^, ^25^, ^26^ ^]^ to promote mesogen self‐assembly into the desired helical structure. However, because the ink viscosity decreases substantially during the annealing process, 3D CLCEs deform and concomitantly lose their structural integrity.

To date, CLCE structures also lack physical memory: when the stimulus – heat, humidity, light, or mechanical strain – is removed, the CLCEs return to their original state. This limits CLCEs to applications as sensors in which stress does not fluctuate. One approach to attain physical memory would be to use bistable structures with multiple stable equilibrium states, i.e., thin‐shell domes. This simple geometry exhibits bistability, i.e., two stable states,^[^ ^29^, ^30^, ^31^ ^]^ but it is difficult to construct without wrinkles when starting with a 2D film. Bistable domes have been used as robotic morphing grippers,^[^ ^30^ ^]^ spatiotemporally programmed surfaces,^[^ ^32^ ^]^ and mechanical sensors with varying electrical resistance.^[^ ^33^, ^34^ ^]^ Their different stable states can lock the triggered stress state and color change in place until the structure is manually reset, resulting in a memory effect. We note that this type of memory is different from shape memory effects, which can hold a deformed shape when mechanical stress and heat are applied simultaneously.^[^ ^15^ ^]^

To fabricate mechanochromic strain sensors with memory, we develop a coaxial DIW method for printing CLCE‐silicone into 3D structures and prepare bistable thin‐shell domes as a demonstration of our method's capabilities. The rheological requirements for DIW of 3D structures are met by utilizing two inks: a fast‐aligning, low‐viscosity CLC ink as the core material and a transparent, yield‐stress silicone ink as the shell material. Coaxially printing these inks allows the CLCE core to be supported by the silicone shell while enabling the cholesteric phase to rapidly self‐assemble as printing ensues. Importantly, both inks are solvent‐free, can be printed at room temperature, and do not require a post‐annealing step. To explore the effects of the silicone shell on CLC alignment and color change, we created core‐shell CLCE elastomer architectures at varying shear rates and characterized their print fidelity, mechanical properties, and mechanochromic sensitivity. Finally, to highlight the versatility of our additive manufacturing platform, we freeform printed monostable domes and conformally printed bistable domes. The bistable domes are arranged into an array capable of strain sensing with memory.

## 2 Results and Discussion

### 2.1 Printable CLC and Silicone Inks

**Figure 1:**
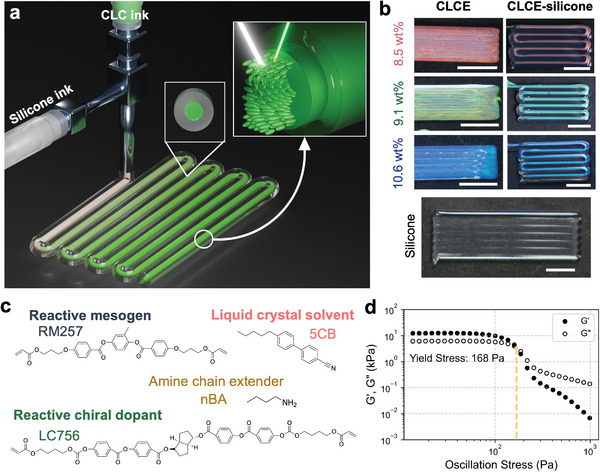
Coaxial 3D printing of silicone‐supported CLCE filament. a) Schematic of the coaxial DIW setup, where the ink is composed of a transparent silicone shell and a CLCE core. Inset: Illustration of CLC alignment within the filament. b) Printed bars of bare CLCE, silicone, and coaxial CLCE‐silicone of various chiral dopant concentrations to achieve red, green, and blue colors. c) Chemical structures of main components in the CLC ink. d) Log‐log plot of the storage modulus, G′ (filled symbols) and loss modulus, G″ (open symbols) as a function of oscillation stress for the silicone ink. Scale bars, 5 mm.

One criterion for DIW is that the inks should be shear thinning, which allows them to be extrudable through fine nozzles. To build 3D structures, the inks also need to possess a sufficiently large shear yield stress to maintain a solid‐like structure under quiescent conditions. To successfully print the low‐viscosity CLC ink, we used a viscoelastic silicone ink to create an encapsulating shell that mechanically supports the CLCE core (**Figure** **1**a,b). In the absence of this silicone shell, the CLCE filaments cannot maintain the structural integrity needed for multilayered architectures. In this configuration, the CLC ink only needs to possess an appropriately low viscosity to promote the desired helical director alignment without the use of an organic solvent or heat. Notably, the viscosities of previously reported CLC inks are greater than 100 Pa s at shear rates of 10^−1^–10^1^ s^−1^ over the temperature range of 25–75 °C,^[^ ^23^, ^26^ ^]^ which greatly hinders director alignment under ambient conditions. To overcome this limitation, we leveraged multiple strategies. First, we synthesized short CLC oligomers via an aza‐Michael addition^[^ ^35^ ^]^ with an acrylate:amine molar ratio of 0.75:1 coupled with a short oligomerization time (50 min). The aza‐Michael addition is chosen for its slower reaction time compared to the thiol‐Michael addition,^[^ ^36^ ^]^ allowing the ink to be printable for several hours without a noticeable change in viscosity. In addition, the chain extender (n‐butylamine) is used as a primary amine (Figure 1c), which results in shorter chains compared to thiol chain extenders. This strategy leads to enhanced mesogen rotation^[^ ^37^ ^]^ thereby diminishing the need for an organic solvent. To further reduce ink viscosity, a nematic small molecule, 4‐cyano‐4′‐pentylbiphenyl (5CB), is used as a diluent. Since 5CB is mesogenic, it does not affect the liquid crystallinity of the mixture.^[^ ^1^, ^5^, ^38^ ^]^ 5CB also does not need to be removed from the ink, since any potential leakage is contained by the silicone shell. Next, excess acrylate monomers are added to the oligomers (final acrylate:amine molar ratio of 1.06:1) to further reduce the ink viscosity and to promote UV crosslinking. Lastly, some mesogenic crosslinker (acrylates) is replaced with a liquid acrylate crosslinker, 1,6‐hexanediol diacrylate (HDDA). The non‐mesogenic properties of HDDA allow for a slight reduction of the ink viscosity while providing some acrylates needed for the UV crosslinking step. To further enhance the color contrast of the CLC inks, 0.1 wt.% carbon black is added to the ink. After combining the oligomers, monomers, and photoinitiators, the ink can then be crosslinked under UV light to form a CLCE. The CLCE color can be blue‐shifted by increasing *c* (Figure 1b), which decreases the pitch and shortens the initial reflected wavelength at *θ*  =  0°, as expected from the HTP equation. In this case, “initial” denotes a state where no stress is applied. This wavelength is referred to as λ_0_.

By contrast, the silicone ink must exhibit a shear‐thinning, viscoelastic response with a sufficiently large shear yield stress to support the CLCE core. It must also be optically transparent to allow the CLCE color to be visible. Silicone is typically cured with heat, but CLCs are heat sensitive and may experience an order‐to‐disorder phase transition or red shift at higher temperatures.^[^ ^14^, ^39^, ^40^ ^]^ Adhesion between the core and shell is also critical to ensure the success of layer‐by‐layer printing. Hence, we use a photocurable silicone ink based on the thiol‐ene chemistry,^[^ ^41^ ^]^ which meets the rheological, optical, and chemical requirements. It is prepared with a commercial polydimethylsiloxane (PDMS) base, Sylgard 186, thiol crosslinkers, and a photoinitiator (Figure S1, Supporting Information). The silicone ink is shear thinning with shear yield stress (τ_y_) of 168 Pa (Figure 1d), which is on the lower end of values (τ_y_ = 101–5000 Pa) reported for other silicone inks,^[^ ^42^, ^43^, ^44^ ^]^ yet sufficient to support multiple layers of CLCE. This ink is also thixotropic and recovers its viscous state within 11.6 s (Figure S2, Supporting Information). Although this recovery is slightly slower than inks presented by Suriboot et al.,^[^ ^45^ ^]^ the loss factor, *G*″/*G*′, where *G*″ is the loss modulus and *G*′ is the storage modulus, reaches a minimum of 0.63 – a *G*″/*G*′ < 0.8 is recommended to prevent slumping.^[^ ^46^ ^]^ The combined yield stress and thixotropic properties of the silicone ink enable the encapsulating shell to fully support the CLCE core during printing. However, the silicone shell does lower the total volume fraction of CLCE in a final printed part and hence, the intensity of reflected light (Figure S3, Supporting Information). Nevertheless, the CLCE color is vibrant and more uniform when printed with a silicone shell (Figure 1d). Using ImageJ, we measure the saturation of the green CLCE‐silicone sample shown in Figure 1b, which is 57.2% for the entire bar and 68.7% for just the CLCE portion, in contrast to literature, 36.2% (for the green CLCE film).^[^ ^23^ ^]^ With our coaxial DIW, we can increase the color saturation beyond this by printing multi‐layered CLCE films.

### 2.2 Optimizing Print Parameters

**Figure 2:**
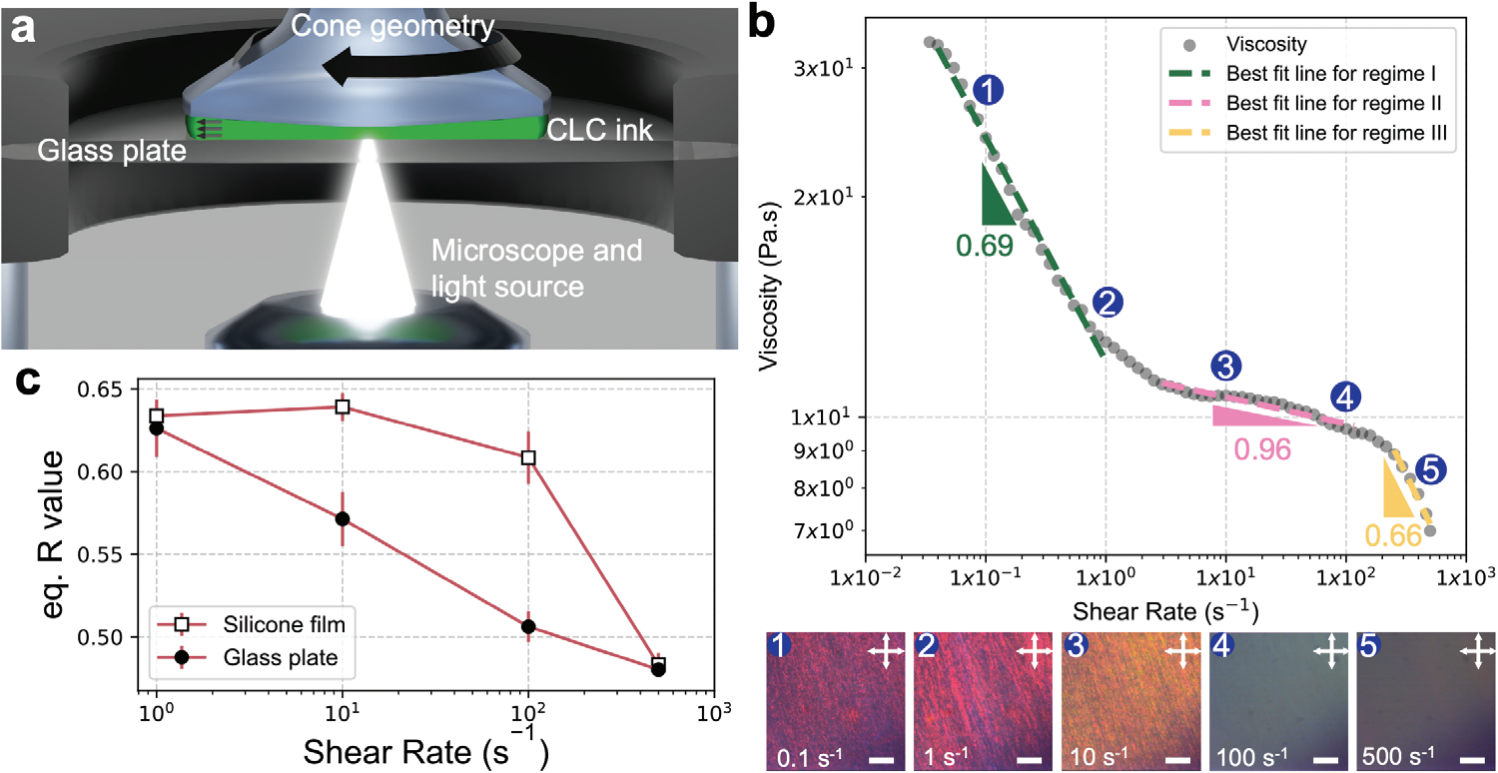
Formation of the cholesteric phase under shear. a) Schematic of the optical rheology setup. b) Viscosity of a red CLC ink as a function of the shear rate, with the power law index indicated for the different shear regimes (top) and polarized optical microscopy (POM) images in reflection mode (viewed through the CLC ink and the glass plate interface) at shear rates of 0.1, 1, 10, 100, and 500 s^−1^ (bottom), respectively. c) Red (from RGB) values of the CLC ink after shearing on a glass plate or a silicone film for 30 s and reaching equilibrium. Scale bars, 100 µm.

The simplest way to increase the reflectance of the CLCE core is to increase the amount of CLC ink being extruded. This translates to increasing the volumetric flow rate if all other printing parameters are the same, leading to an increase in shear rate (see Supporting Information Text) – which affects CLC alignment. To determine how the CLC alignment is affected by shear rate, we shear a red CLC ink on a glass plate and observe temporal changes to color using a rheometer equipped with in‐situ polarized optical microscopy (POM) in the reflection mode (**Figure** **2**a; Figure S4, Supporting Information). The viscosity curve indicates that there are three flow regimes, as seen in some nematic LCE inks,^[^ ^47^, ^48^ ^]^ representing different power‐law indices, *n* (Figure 2b). The indices are used to calculate the wall shear rate, ${\dot{\gamma }}_{\mathrm{CLC}}$, generated from various printing pressures (see Supporting Information Text). The CLC ink is sheared for 30 seconds at a designated shear rate (0.1, 1, 10, 100, or 500 s^−1^) and then shear is stopped to observe the cholesteric formation over time (Movies S1 and S2, Supporting Information). The POM images in Figure 2b are taken at the end of the 30 s shearing stage. We will first discuss the alignment during the shearing stage. For ${\dot{\gamma }}_{\mathrm{CLC}}$ = 0.1 s^−1^, the ink looks red under POM, which signifies the presence of the cholesteric phase. There is negligible movement or color change in the ink, meaning the ink is already cholesteric without any shear. However, ink that has not been sheared appears polydomain at a macroscopic level, implying that at least 0.1 s^−1^ of shear is required to generate uniform alignment. For ${\dot{\gamma }}_{\mathrm{CLC}}$ = 1 s^−1^, the ink still reflects red, but the color intensity is greater than that of the 0.1 s^−1^ shear–indicating the formation of a more monodomain structure. At ${\dot{\gamma }}_{\mathrm{CLC}}$ = 10 s^−1^, the color starts to shift to orange, suggesting that the cholesteric phase is still present but now slanted.^[^ ^22^ ^]^ At 100 and 500 s^−1^, bright and dark flashes are seen during the shearing stage, indicating that a flow‐induced nematic phase is present^[^ ^49^ ^]^ instead of the CLC phase (Figure S5, Supporting Information). Although the nematic phase is present during ${\dot{\gamma }}_{\mathrm{CLC}}$ = 100 and 500 s^−1^, the cholesteric phase quickly forms upon flow cessation. Characterizing the presence of different phases during the shearing stage helps us understand and control the formation of the CLC phase after shear is stopped.

To quantify the relative LC ordering due to ${\dot{\gamma }}_{\mathrm{CLC}}$, the average red value (from RGB) is calculated after the color stabilizes. This is referred to as the red equilibrium value, *R*_eq_. To visualize the colors during and after shear, the RGB values are extracted from each frame and mapped in Figure S6 (Supporting Information). Numerical RGB values are plotted against time in Figure S7 (Supporting Information). The *R*_eq_ is found to be highest for ${\dot{\gamma }}_{\mathrm{CLC}}$ = 10 s^−1^ and decreases with increasing shear rate. The time to reach equilibrium, *t*_eq_, is determined as the time at which the average *R* value of following frames is within 0.05% of *R*_eq_. The *t*_eq_ is quickest at 500 s^−1^ but *R* is the lowest, meaning the alignment is lowest at 500 s^−1^. *R*_eq_ and *t*_eq_ values are summarized in **Table** **1**. The *t*_eq_ is likely fastest at ${\dot{\gamma }}_{\mathrm{CLC}}$ = 500 s^−1^ because the nematic phase is obtained during shear (regime III in Figure 2b), which is an entropically stable state for LCs. After shear stops, twisting occurs (due to the chiral dopant), but the difference in entropy between the cholesteric and nematic phases may not be great enough to overcome the viscosity recovery, thus resulting in a frozen intermediate phase in the innermost ink (farthest from the cone and plate). POM images of the inks at *t*_eq_ and various shear rate are shown in Figure S8 (Supporting Information). In coaxial DIW of CLCE‐silicone, the CLCE is in contact with silicone rather than with glass, so we repeat the shear test on a silicone‐coated glass plate to verify the effect of silicone on *R*_eq_ and *t*_eq_. Compared to the control experiment on glass, the *R*_eq_ on silicone generally increases (Figure 2c) and the *t*_eq_ generally decreases, which indicates that the silicone film improves the CLC alignment. Looking only at the values of the test performed on silicone, the highest *R*_eq_ value is found at 10 s^−1^, which also results in the second fastest *t*_eq_. Although the next highest *R*_eq_ value is found at ${\dot{\gamma }}_{\mathrm{CLC}}$ = 1 s^−1^, the *R*_eq_ value for ${\dot{\gamma }}_{\mathrm{CLC}}$ = 100 s^−1^ is not very different and the *t*_eq_ values are similar as well. Thus, based on *R*_eq_ and *t*_eq_, the optimal range of shear that can be used to print CLCE‐silicone is between 1 and 100 s^−1^. ${\dot{\gamma }}_{\mathrm{CLC}}$ = 0.1 s^−1^ is not used for these comparisons because the color changes minimally throughout the test, making it difficult to gauge when equilibrium is reached (Figure S6, Supporting Information).

**Table 1:** Summary of the equilibrium time, *t*_eq_, and the equilibrium red (from RGB) values, *R*_eq_, of a red CLC ink sheared at various shear rates.

	Glass		Silicone	
Shear rate	*t*_eq_ [s]	*R*_eq_	*t*_eq_ [s]	*R*_eq_
1 s^−1^	30.9 s	0.64	28.3 s	0.64
10 s^−1^	21.8 s	0.57	22.0 s	0.65
100 s^−1^	33.0 s	0.51	27.7 s	0.61
500 s^−1^	15.6 s	0.49	14.4 s	0.48

**Figure 3:**
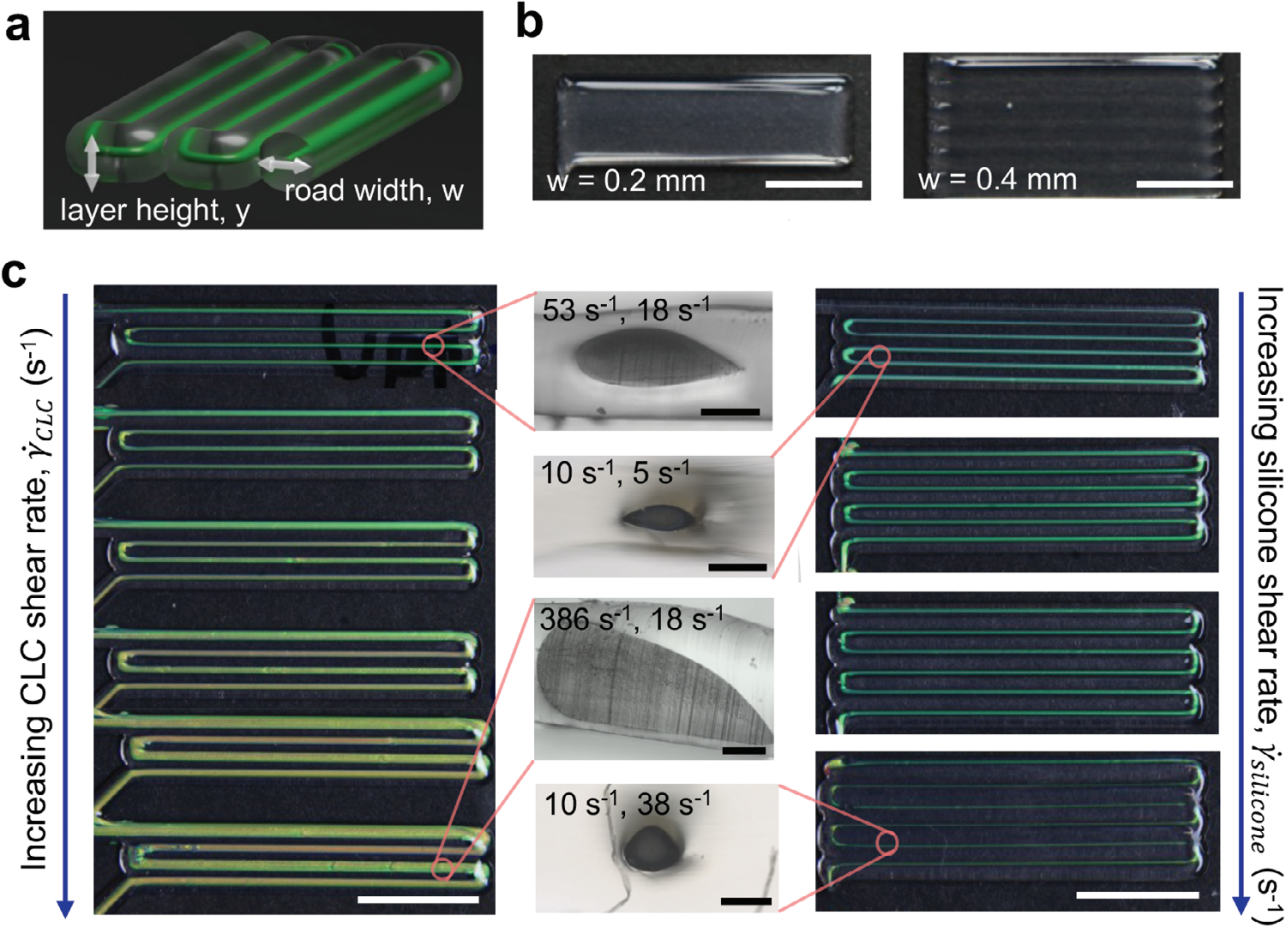
Shear rate effects on print fidelity. a) Schematic of a CLCE‐silicone bar with a layer height, *y*, and road width, *w*. b) Silicone bars printed with different road widths, using a 22‐gauge needle (0.406 mm inner diameter). c) Left and right: optical images of the CLCE‐silicone bars printed with *y* = 0.4 mm, *w* = 1.4 mm, at a constant silicone shear rate ${\dot{\gamma }}_{silicone}$ = 18 s^−1^ and increasing CLCE shear rate ${\dot{\gamma }}_{\mathrm{CLC}}$ (left), and CLCE‐silicone bars printed with *y* = 0.4 mm, *w* = 1.4 mm, ${\dot{\gamma }}_{\mathrm{CLC}}=$ 85 s^−1^ and increasing ${\dot{\gamma }}_{silicone}$ (right). Scale bars, 5 mm. Middle: transmission microscopy images of the cross‐section of the CLCE‐silicone bars with shear rates ${\dot{\gamma }}_{\mathrm{CLC}}$, ${\dot{\gamma }}_{silicone}\;$ Indicated, respectively. Scale bars, 400 µm.

The effects of the CLCE and silicone ink shear rates are also evident on a macroscopic scale. To be consistent, we printed the CLCE‐silicone bars with a nozzle speed of 120 mm min^−1^, similar to those used in literature.^[^ ^7^, ^9^, ^10^ ^]^ Due to the low viscosity of the CLC ink, a slanted helical axis is not observed at this nozzle speed. The longest reflected wavelength is observed normal to the print surface and a blue shift in color is observed when printed bars are viewed at different angles (Figure S9, Supporting Information). Layer height, *y*, is chosen such that the instabilities that arise from over and under extruding are mitigated. The road width, *w*, (**Figure** **3**a) is chosen by eye to minimize gaps between rows. Increasing *w* leads to reduced overlap, causing the interstitial spaces to scatter light and thus resulting in a less uniform sample. This is most evident in a printed silicone sample (Figure 3b). When *w* is too large in a CLCE‐silicone print, the increased scattering results in glare and obstructs the reflected CLCE colors. The CLCE‐silicone samples printed with higher ${\dot{\gamma }}_{\mathrm{CLC}}$ have a longer pitch and appear more inhomogeneous (Figure 3c). Increasing ${\dot{\gamma }}_{\mathrm{CLC}}$ is achieved by increasing the volumetric flow rate while maintaining the same nozzle speed, which extrudes more CLC ink and leads to filaments with higher CLCE to silicone volume ratio. As seen in the cross‐sectional geometry of samples printed with the same *y* and *w*, this leads to an increasingly asymmetrical core. The CLC ink starts to sink within the silicone because the silicone has a slightly lower density (1.088 g cm^−3^ vs 1.136 g cm^−3^) and cannot support large amounts of CLCE. Since the bars are printed with the same layer height and road width, a larger core also leads to more overlap between the rows. The silicone from the newly printed row squeezes the previous rows, resulting in a deformed teardrop shape (Figure 3c). If a more symmetric core is desired, we can increase ${\dot{\gamma }}_{\mathrm{silicone}}$ and decrease ${\dot{\gamma }}_{\mathrm{CLC}}$ to provide a greater distance between the CLCE cores, but the cores end up cylindrical and appear thinner than the teardrop‐shaped cores from the top, making it more difficult to view the color (Figure 3c). The flatter, ellipsoidal geometries are also better for printing multiple layers, as discussed later. We therefore find the optimal printing conditions for multilayer and vibrant CLCE‐silicone multi‐filament thick films arise from a silicone shear rate of 5–26 s^−1^ when *w* = 0.4 mm. From these results, we can also refine the optimal range of ${\dot{\gamma }}_{\mathrm{CLC}}$ further to be 10–100 s^−1^ since we can tell from the CLCE‐silicone bars printed with ${\dot{\gamma }}_{\mathrm{CLC}}$ = 10 s^−1^ that further decreasing ${\dot{\gamma }}_{\mathrm{CLC}}$ will make the color difficult to see. We can also observe from printing that utilizing a silicone shell prevents defects from growing. When CLC ink is printed by itself, rows bleed into each other, and air bubble defects spread on the glass to become larger defects (Figure S10, Supporting Information). When CLC ink is printed inside silicone, the air bubbles do not spread due to the non‐wetting behavior of CLC ink on silicone, which has a lower surface energy. This is confirmed by measuring the contact angle of the CLC ink on glass (25.18 ± 1.72°) and on silicone (64.81 ± 0.99° for uncured silicone and 56.64 ± 0.54° for cured silicone). The contact angle of our CLC ink on glass is similar to that reported in literature (22°),^[^ ^27^ ^]^ using the same LC monomer and chiral dopant (RM257 and LC756). The contact angle of ink on uncured silicone is the largest of the three substrates (Figure S11, Supporting Information), which indicates that the enhancement of CLC alignment inside the uncured silicone shell is due to non‐wetting behavior driving the mesogens to assemble into a favorable arrangement: the cholesteric phase.

**Figure 4:**
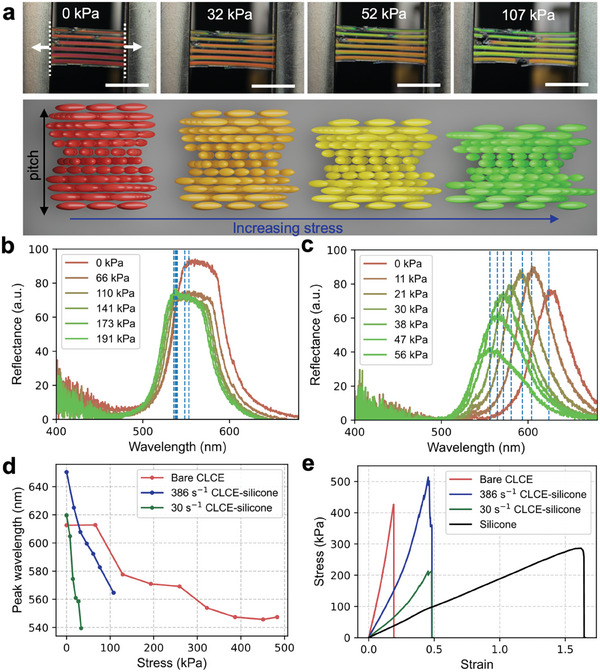
Mechanochromic properties of the printed CLCE‐silicone bars. a) Optical images of a red CLCE‐silicone bar under uniaxial tension and the corresponding illustrations of the helix deformation. Scale bars, 5 mm. b) Reflectance spectra of the red CLCE as a function of stress. c) Reflectance spectra of the red CLCE‐silicone as a function of stress. d) Peak wavelengths of the bare CLCE and CLCE‐silicone bars as a function of stress. e) Stress–strain curves of the bare CLCE, silicone, and CLCE‐silicone bars (with ${\dot{\gamma }}_{\mathrm{CLC}}\;$ = 386 and 30 s^−1^) under uniaxial tension.

Next, we investigated the mechanochromic properties of the printed CLCE‐silicone bars. To characterize the mechanochromism, the samples are loaded into a tensile tester with a spectrometer probe pointing normally to the sample surface (Figure S12, Supporting Information). Uniaxial tensile testing is performed at 5 mm min^−1^ while the spectrometer reads at intervals of 0.5 s. As seen in **Figure** **4**a–c, λ_0_ increases with increasing ${\dot{\gamma }}_{\mathrm{CLC}}$ and cured prints show a blue shift when tensile stress is applied. The blue shift is caused by lateral compression accompanying uniaxial tension, which reversibly compresses the pitch. The reduction in pitch is accompanied by reversible deformation of the helical structure, leading to a reduction in reflectance.^[^ ^17^, ^50^ ^]^ The mechanochromic sensitivity is defined as where is the shortest peak wavelength measured and is the corresponding stress at this wavelength. Bare CLCE exhibits a sensitivity of 0.09 nm kPa^−1^ whereas the CLCE‐silicone sensitivity is higher and ranges from 0.79–2.4 nm kPa^−1^ (Figure 4d). As ${\dot{\gamma }}_{\mathrm{CLC}}$ increases, the mechanochromic sensitivity decreases (**Table** **2**), which further agrees with the optimal range of ${\dot{\gamma }}_{\mathrm{CLC}}$ = 10–100 s^−1^. Cyclic tensile testing of a green CLCE‐silicone bar with ${\dot{\gamma }}_{\mathrm{CLC}}$ = 85 s^−1^ shows that a blue shift in color is visible by eye and reversible for at least 100 cycles under 20% strain. At 30% and 40% strain, some plastic deformation occurs (Figure S13 and Movie S3, Supporting Information). Therefore, 20% strain is chosen for cyclic testing.

**Table 2:** Summary of mechanochromic sensitivity of the bare CLCE and the CLCE‐silicone printed bars at different CLCE shear rates.

	Mechanochromic sensitivity [nm kPa^−1^]
CLCE	0.13
30 s^−1^	2.39
75 s^−1^	1.87
110 s^−1^	1.35
156 s^−1^	1.21
386 s^−1^	0.79

We also examined the mechanical properties of CLCE, silicone, and CLCE‐silicone printed bars (Figure 4e) to validate the role of the silicone shell. The cured silicone has Young's modulus of 202 kPa with a fracture strain of 165%. The cured CLCE is stiffer and less elastic than silicone with a Young's modulus of 1.647 MPa and fracture strain of 19.1%. The Young's modulus and fracture strain of CLCE‐silicone depend on ${\dot{\gamma }}_{\mathrm{CLC}}$ since it influences the CLCE volume fraction (if all other print conditions are kept the same). Accordingly, increasing ${\dot{\gamma }}_{\text{CLC}}$ results in an increased Young's modulus of the CLCE‐silicone print. Thus, the samples printed at a larger ${\dot{\gamma }}_{\mathrm{CLC}}$ equire more applied force to reach the same magnitude of wavelength shift as those printed at lower shear rates (Figure 4d; Figure S14, Supporting Information). The CLCE‐silicone prints all have a much greater fracture strain (48.2–69.5%) than the pristine CLCE samples (**Table** **3**), which is attributed to the softer and more elastic silicone shell. The poor elasticity of the CLCE is a tradeoff from the short oligomerization time and non‐reactive 5CB, however, the silicone shell mitigates some of the negative effects.

**Table 3:** Young's modulus and fracture strain of the CLCE, silicone, and CLCE‐silicone bars.

	Young's modulus [kPa]	Fracture strain [%]
CLCE	1647	19.1
Silicone	202	165
CLCE‐silicone (30/18)	338	49.1
CLCE‐silicone (75/18)	375	60.3
CLCE‐silicone (110/18)	472	53.8
CLCE‐silicone (157/18)	468	69.5
CLCE‐silicone (386/18)	748	48.2

* Indicating the CLCE and silicone shear rates in s^−1^.

### 2.3 Printing 3D CLCE‐Silicone Structures

**Figure 5:**
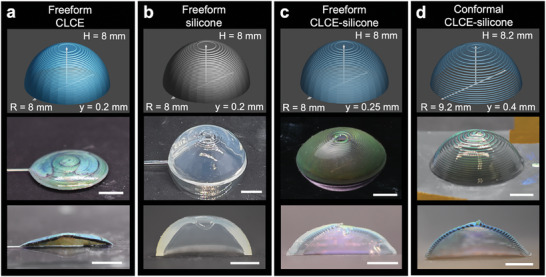
Domes printed from CLCE, silicone, and CLCE‐silicone, respectively. Top panels: print paths of domes with radius *R*, height *H*, and layer height *y*. Middle panels: optical images of the representative samples. Bottom panels: optical images of the cross‐sections of a) a bare CLCE printed without a substrate (freeform), b) freeform silicone, c) freeform CLCE‐silicone, and d) conformally printed CLCE‐silicone. Scale bars, 5 mm.

The pristine CLC ink, when printed by itself in more than two layers, starts to lose its shape and resolution (**Figure** **5**a). The silicone ink can be printed into hollow 3D structures on its own (Figure 5b) because of its yield stress. The support from the silicone shell allows us to print a multi‐layer CLCE‐silicone cylinder (Figure S15, Supporting Information). For ease of comparison, the same nozzle size (22–17 gauge, or core diameter = 0.406 mm, shell thickness = 0.180 mm) is used for all samples. The success of a multi‐layer print depends on the layer height, as too large of a print height can cause the cylinder to collapse in the middle. This happens because the silicone cannot recover its viscous state fast enough to maintain a cylindrical cross‐section while more layers are being printed on top, so gravity pulls the layers in toward the center of the cylinder, resulting in a hyperbolic shape. This may be mitigated by printing much slower or utilizing in‐situ UV‐curing, however, this will start to cure the CLCE core before *t*_eq_ is reached, so either different photoinitiators could be used or the alignment and curing speeds could be tuned and optimized. Using a smaller layer height of 0.25 mm to obtain flatter filaments results in a successful print of a 5 mm tall cylinder (Figure S15, Supporting Information). We can see that the top of the cylinder is also starting to cave in, which is likely due to the viscoelastic nature of the CLC ink, causing more ink to flow after a few minutes of continuous extrusion. This could be fixed by increasing the yield stress of the silicone ink, by periodically stopping the CLC ink flow, or by decreasing ${\dot{\gamma }}_{\text{CLC}}$ at increasing print times.

The effect of gravity can also be seen when we attempt to print freeform thin‐shell dome structures. First, we attempt to print CLCE‐silicone domes without the use of a substrate. Due to gravity, printing a shell in a freeform manner results in domes with increasing thickness toward the base, rather than a consistent thickness throughout the cross section (Figure 5c). This makes a dome monostable: the inverted state is unstable, and the dome quickly snaps back to its original state when the external force is removed (Movie S5, Supporting Information). In order to successfully print a complete dome without the use of a substrate, a small layer height (*y* = 0.25 mm) is utilized. This causes the layers, and thus the CLCE cores, to be flattened (Figure 5c). The flattened cores show an obvious angle dependence of the color that is similar to CLCE films. As the angle of incidence of a dome in its unstressed state increases from 0° (top view) to 90° (side view), we see a blue shift in color. The tensile stress at the bottom face of an inverted, initially red dome causes a blue shift as well, as seen in the green spot on the face (Figure S16, Supporting Information). When inverted, a majority of the dome loses its color because the flat CLCE cores are turned sideways, allowing light to transmit through (similar to window blinds). The inverted dome lip also blue shifts to green due to the hoop stress.

**Figure 6:**
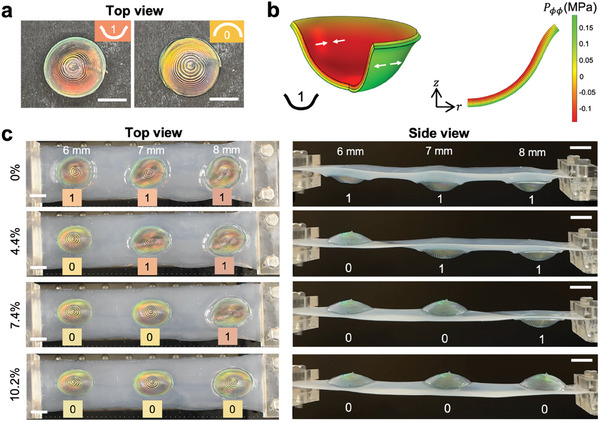
A passive and untethered mechanochromic sensor array with memory. a) Top‐view photos of a conformally printed CLCE‐silicone dome. The numbers, 1 and 0, indicate whether the dome is inverted or not, respectively. The colors of the insets represent the colors of the dome centers. Scale bars, 5 mm. b) Simulation of the compressive and tensile stresses (indicated with white arrows) applied to an orange CLCE‐silicone dome under inversion, with red depicting compression and green depicting tension. The legend indicates the magnitude of engineering stress on an inverted dome, with negative values for compression and positive values for tension. c) Three domes printed on dome substrates of the same radius (8 mm) but different heights (6–8 mm) are arranged on a silicone sheet as the substrate for stretching, which is viewed from the top (left) and side (right) to track the dome reversions at different strains. Scale bars, 1 cm.

To minimize the effect of gravity on the CLCE‐silicone and achieve bistable domes with clearly visible color change, we then conformally print the CLCE‐silicone on a domed substrate (Figure 5d), which allows us to print steeper overhangs and thinner domes with uniform thickness. To prepare the domed substrate, we use fused deposition modeling (FDM) with acrylonitrile styrene acrylate (ASA) filament and vapor smooth the prints. A smooth substrate helps minimize scattering from the silicone shell since the silicone will pick up the detail from the visible layer heights. Substrates can also be printed via stereolithography (SLA) and then sanded. We find that the smoothest substrates are obtained with the combination of FDM and vapor smoothing. The CLCE‐silicone domes can be carefully peeled off the substrate after curing. Since there is no organic solvent in the CLCE and silicone inks and the ASA filament is UV‐resistant, the substrates are not damaged and can be reused many times. The conformally printed CLCE‐silicone domes have a more uniform thickness compared to the domes that were printed without a substrate (Figure 5c,d). However, there is also increased iridescence due to the difficulty in perfectly centering the print on the substrate (Figure S17, Supporting Information). A possible solution for this is to fabricate molds with guides for the nozzle starting position. Color nonuniformity is also due to the residual stresses from printing and curing on a substrate, but perfectly centering the print on the substrate would minimize these defects. For this reason, it is inadvisable to print CLCE directly onto the substrate since the silicone also acts as a buffer between the CLCE and substrate. Compared to the freeform domes, the conformal domes can be printed accurately into domes with curvatures of 0.12 mm^−1^ and are thin and tall enough to exhibit bistability.^[^ ^51^ ^]^ Thus, the two stable states (0 and 1, corresponding to the base and inverted configurations respectively) can be locked in without requiring additional energy to maintain them (**Figure** **6**a). When a dome is inverted, the top face undergoes compression (red shift in the color) whereas the bottom face undergoes tension (blue shift). For a dome printed on a substrate with radius *R* = 8 mm and height *H* = 8 mm, finite element analysis (FEA) shows that when the dome is inverted, the bottom and top faces experience a maximum tangential tensile stress of 0.189 MPa and a maximum compressive stress of 0.140 MPa, respectively (Figure 6b). The difference in the type of stress results in different visible color shifts when the inverted domes are viewed from the top versus the bottom (Figure 6a; Figure S18, Supporting Information). The difference in color on the two faces is another degree of freedom that thin CLCE films do not exhibit. Some CLCE films are so soft that a backing layer (typically colored black for maximum CLCE reflection) is needed, which prevents color from being seen from both sides. Although freestanding CLCE films also experience tensile and compressive stresses when bent, the nature of the planar geometry makes it difficult to see the face undergoing compression. The dome dimensions also influence the mechanochromic response. Theoretically, decreasing the dome height decreases the magnitude of stress imparted on the dome in its inverted state, leading to a smaller shift in color. Experimentally, the domes show the same trend such that shorter domes experience a smaller blue shift under compression (Figure S19, Supporting Information). More significant differences in stress and the corresponding color change could be achieved by also varying the dome thicknesses. To highlight how to fabricate a strain sensor with memory, we arrange domes printed on molds of the same radius (8 mm) and varying heights (6, 7, and 8 mm) into an array and connect them with a UV‐cured silicone film. The dimensions of the domes are presented in Table S1 (Supporting Information). The film is then clamped in a linear stage and the domes are inverted to state 1 by manually pressing them (Movie S5, Supporting Information). Since the domes are bistable, they maintain state 1 (red) until tension is applied. When an increasing strain is applied uniaxially to the array, the domes begin to revert to state 0 (yellow), from shortest to tallest dome sequentially, where the increasing height corresponds to an increasing energy barrier to overcome for snap‐through (Movie S6, Supporting Information). The snap‐through acts as the “save” function, such that the array can remember the strains at 4.4, 7.4, and 10.2%, respectively (Figure 6c). Even if the applied strain is removed, the domes remain in their saved states and corresponding colors. Thus, the maximum strain level reached by the sensor is stored in the mechanochromic memory of the sensor. For example, if the array is stretched and released before the tallest dome reverts (strain >7.4% and <10.2%), the first two domes stay in the same reverted stable state 0. Stretching the array cannot invert the domes from state 0 to state 1, which indicates that state 1, though stable, exists at a higher energy minimum than state 0. In this array, the domes are spaced one diameter length (16 mm) apart, which is sufficient to prevent one dome reversion from triggering another. Using different distances and non‐linear patterns would change the snap‐through responses.^[^ ^30^ ^]^ If different patterns are combined with domes of different heights and radii, the response could potentially be tuned such that only the right amount of stress will decode a message–which may have possible uses in cryptography and anti‐counterfeiting. Other multi‐stable structures could also be printed to generate arrays with greater memory storage.

## 3 Conclusion

In summary, we established a coaxial DIW method for fabricating 3D CLCE‐silicone structures. The CLCE and silicone inks are synthesized without the use of organic solvents and do not require an additional annealing step for the cholesteric phase to form. The silicone shell not only supports the CLCE core to enable layer‐by‐layer printing, but it also increases the elasticity and mechanochromic sensitivity of the CLCE. As an exemplar, we printed bistable mechanochromic domes and arranged them into a strain sensor capable of physical memory that is sequentially activated at 4.4%, 7.4%, and 10.2% strain. Looking ahead, coaxial DIW offers a facile platform for patterning mechanochromic inks in the form of logic gates, microactuators, and gradient colors for potential applications in smart wearables, soft robotics, and cryptography.

## 4 Experimental Section

### Materials

LC monomers 1,4‐bis‐[4‐(3‐acryloyloxypropyloxy)benzoyloxy]‐2‐methylbenzene (RM257, 98%) and 4‐cyano‐4′‐pentylbiphenyl (5CB, 98%) were purchased from AmBeed. Chiral dopant monomer, hexahydrofuro[3,2‐b]furan‐3,6‐diylbis(4‐((4‐(((4‐(acryloyloxy)butoxy)carbonyl)oxy)benzoyl)oxy)benzoate) (LC756, 97%) was purchased from Chemfish Tokyo and Synthon. Chain extender, n‐butylamine (nBA, 99.5%), inhibitor, butylated hydroxytoluene (BHT), and non‐mesogenic monomer, 1,6‐hexanediol diacrylate (HDDA, 80%) were purchased from Sigma‐Aldrich. Carbon black (Super P Conductive, >99%) was purchased from Alfa Aesar. Photoinitiators, phenyl bis(2,4,6‐trimethylbenzoyl)phosphine oxide (I‐819, 97%), 2,2‐dimethoxy‐2‐phenylacetophenone (I‐651, 99%), and 2‐hydroxy‐2‐methylpropiophenone (I‐1173, 97%) were purchased from Sigma‐Aldrich. Sylgard 186 was purchased from Dow Corning. Crosslinkers, [4‐6% (mercaptopropyl)methylsiloxane] – dimethylsiloxane copolymer (SMS‐042) and [13‐17% (mercaptopropyl)methylsiloxane] – dimethylsiloxane copolymer (SMS‐142), were purchased from Gelest. All chemicals were used as received.

### CLC Ink Preparation

The CLC ink was prepared by combining CLC oligomers and a monomer mixture, resulting in a reactive CLC ink with an acrylate:amine molar ratio of 1.06:1. The CLC oligomers were first prepared by melting RM257, LC756, and 1 wt.% BHT in a 90 °C oven. For red, green, and blue CLC inks, LC756 made up 8.5, 9.1, and 10.6 wt.% of the total LC content, respectively. The total LC content included the RM257 and LC756 used to prepare the ink as a whole (oligomers and monomers). Carbon black was added to 5CB at 0.1 wt.% and sonicated for 15 min before the mixture was added to the melted monomers at a 1:2 (5CB:total LC monomer) mass ratio. nBA was then added to make a total acrylate:amine molar ratio of 0.75:1 (acrylates from RM257 and LC756), and the mixture was left to oligomerize in a 60 °C oven for 50 min. To prepare the CLC ink for printing, a mixture of RM257, LC756, HDDA, 0.5 wt.% I‐819, 1 wt.% I‐651, and 0.5 wt.% BHT was melted in a vial in a 90 °C oven. The HDDA:amine molar ratio was 0.025:1. CLC oligomers were then added to this monomeric mixture to make a final acrylate‐to‐amine molar ratio of 1.06:1. Here, acrylate content included the oligomers, monomeric RM257, monomeric LC756, and monomeric HDDA.

### Silicone Ink Preparation

Sylgard 186 (89 wt.%), SMS‐042 (9 wt.%), SMS‐142 (1 wt.%), and I‐1173 (1 wt.%) were added to a container and mixed for 30 s at 2000 rpm and defoamed for 30 s at 2200 rpm using a centrifugal mixer (Thinky, ARE‐310). The density of silicone ink was calculated by loading the ink into a 1 mL syringe, centrifuging the syringe, and extruding the ink onto a balance (Sartorius, BCE223‐1S).

### Printer Setup

The inks were prepared, loaded into syringes, and centrifuged to remove air bubbles. A 22‐17‐gauge coaxial needle (ramé‐hart) was used for printing. Printing was performed with a nozzle speed of 120 mm min^−1^ at 25 °C. A custom 3‐axis stage motion control stage (Aerotech) equipped with a pressure box (Nordson) and a Hyrel 30M were used for controlling the print movement. The open‐source MeCode was used to produce G‐code for the Aerotech, and a custom Python script was used to produce G‐code for the Hyrel. Prints fabricated with the Aerotech were cured in a chamber (XYZprinting, UV Curing Chamber) at level 3 for 20 min. Prints fabricated with the Hyrel were cured with a handheld 365 nm UV light (Thorlabs, CS20K2) for 6 min. The CLCE‐silicone bars for characterization were printed with the Aerotech setup and the CLCE‐silicone domes were printed with the Hyrel setup. The CLC ink shear rates were calculated by measuring the mass flow rate at different extrusion pressures (Figure S20, Supporting Information). The domed substrate design (array of three domes with radii 8 mm and 6, 7, and 8 mm heights) for conformal printing was prepared in SolidWorks. UltiMaker Cura was used to slice the file and UltiMaker 2+ was used to print the substrate using ASA filament (Polylite ASA, Polymaker). The layer height was 0.1 mm and the print speed was 60 mm s^−1^. Then, vapor smoothing with acetone was performed in 15 min intervals until the layer lines were not visible.

### Rheology and Color Analysis

Optical rheology was performed with a TA Instruments Discovery Hybrid Rheometer with a 20 mm 1° cone geometry and gap height of 29 µm. A glass plate (TA instruments, Modular Microscope Accessory) was used so that a camera (Imaging Development Systems, UI‐3250CP) could record the ink through the glass (Figure S4, Supporting Information). Crossed polarizers were added to the light path to perform POM. All videos were taken with the same white balance, exposure time, and frame rate. The videos were then analyzed with openCV to extract RGB values, which were normalized by dividing by 255. The silicone‐coated glass was prepared by depositing silicone ink onto a glass slide, sandwiching the ink with another glass slide, and curing. The second glass slide was then removed.

### Mechanochromic Characterization

Tensile testing was performed with an Instron 5564 Tabletop Universal Testing Machine equipped with a 10 N load cell. The extension speed was 5 mm min^−1^ and the strain is defined relative to the initial length. Cyclic testing was performed at 5 mm min^−1^. For mechanochromic characterization, a bifurcated reflection probe connected to a spectrometer (OceanOptics) and broadband light source (Thorlabs SLS201L) was aimed at the sample 5 mm away to measure the reflectance during tensile testing at intervals of 0.5 s.

### Optical Microscopy

Optical microscopy was performed on a Keyence VHX‐7000 using bright field transmission.

### Finite‐Element Analysis

Finite‐element analysis was prepared in COMSOL to calculate the stress in a conformally printed CLCE‐silicone dome in the inverted state, using quadrilateral elements with axial symmetry. For simplicity, the CLCE‐silicone material was modeled as an isotropic, linear elastic material with Young's modulus 748 kPa, corresponding to ${\dot{\gamma }}_{\mathrm{CLC}}/{\dot{\gamma }}_{\mathrm{silicone}}$ = 386 s^−1^/18 s^−1^. The dome dimensions were radius 9.15 mm, height 8.2 mm, and thickness 0.985 mm (Figure 6b).

### Optical Imaging

Optical images and videos were taken with a Nikon D5600 camera and Nikon AF‐S DX NIKKOR 18–55 mm f/3.5‐5.6G VR lens.

### Contact Angle

The contact angle of CLCE precursor on various surfaces was found by utilizing a dispenser (Nordson Ultimus I) and a goniometer (ramé‐hart Model 250). ImageJ was used to measure the contact angle.

## Conflict of Interest

The authors declare no conflict of interest.

## Author Contributions

A.N., K.S.R., C.C.C., E.L., and S.Y. conceived the research ideas; A.N. prepared the materials and conducted the rheological, mechanical, and colorimetric characterizations; A.N. conducted the coaxial DIW with the help of R.T.; R.T. extracted the color values from the POM videos; K.S.R. conducted the simulations; A.N. and S.Y. wrote the manuscript; C.C.C., E.L., J.A.L., and S.Y. supervised the research; all authors discussed the results and reviewed the manuscript.

## Supporting information



Supporting Information

Supplemental Movie 1

Supplemental Movie 2

Supplemental Movie 3

Supplemental Movie 4

Supplemental Movie 5

Supplemental Movie 6

## Data Availability

The data that support the findings of this study are available in the supplementary material of this article.
