# Supplementary material for: Coaxial Direct Ink Writing of Cholesteric Liquid Crystal Elastomers in 3D Architectures
Source: Adv Mater. 2025 Jan 26;37(10):2416621. doi: 10.1002/adma.202416621 (PMC11899511; doi:10.1002/adma.202416621)
Supplement: Supplementary file 1 — Supporting Information [file ADMA-37-2416621-s002.pdf]

ADVANCED MATERIALS

Supporting Information

for *Adv. Mater.*, DOI 10.1002/adma.202416621

Coaxial Direct Ink Writing of Cholesteric Liquid Crystal Elastomers in 3D Architectures

Alicia Ng, Rodrigo Telles, Katherine S. Riley, Jennifer A. Lewis, Caitlyn C. Cook, Elaine Lee* and Shu Yang**

Supporting Information

Coaxial Direct Ink Writing of Cholesteric Liquid Crystal Elastomers in 3D Architectures

Alicia Ng, Rodrigo Telles, Katherine S. Riley, Jennifer A. Lewis, Caitlyn C. Cook, Elaine Lee*, Shu Yang**

E-mail: krikorian3@llnl.gov, lee1040@llnl.gov, shuyang@seas.upenn.edu

Wall shear rate for the CLCE core. The core shear rate can be calculated according to

$$\dot{\gamma}_w = \left(\frac{3n+1}{4n}\right)\frac{32\dot{Q}}{\pi D_c^3} \quad \text{(S1)}$$

where n is the power law index fitted from viscosity data (slope = $n-1$), $\dot{Q}$ is volumetric flow rate, and D_c is nozzle diameter of core. $\dot{Q}$ is calculated by measuring the mass flow rate, $\dot{m}$, at various extrusion pressures and dividing by density, ρ. The CLC ink density is taken as the weighted average of the density of an LC ink[1] and the density of 5CB. Thus, ρ = 1.136 g cm^3. A 22-gauge nozzle is used for the core, which has an internal diameter (ID) of 0.406 mm.

Annular shear rate for the silicone shell. Following approach from Bird et al.[2].

If $\kappa \leq \xi \leq \beta$

$$v_z(\xi) = \frac{\dot{Q}(s+3)}{\pi R^2\{(1-\beta^2)^{1+s} - \kappa^{1-s}\cdot(\beta^2-\kappa^2)^{1+s}\}}\int_\kappa^\xi \left(\frac{\beta^2}{z} - z\right)^s dz \quad \text{(S2)}$$

If $\beta \leq \xi \leq 1$

$$v_z(\xi) = \frac{\dot{Q}(s+3)}{\pi R^2\{(1-\beta^2)^{1+s} - \kappa^{1-s}\cdot(\beta^2-\kappa^2)^{1+s}\}}\int_\kappa^\xi \left(z - \frac{\beta^2}{z}\right)^s dz \quad \text{(S3)}$$

where $s = 1/n$, $\kappa = \frac{core_{OD}}{shell_{ID}}$, and $R = \frac{shell_{ID}}{2}$. The power law index, n, is fitted from viscosity data (Figure S21). A 17-gauge nozzle is used for the shell, which has an ID of 1.06 mm. The core outer diameter (OD) is found from the 22-gauge nozzle, which is 0.711 mm.

References

[1] Kotikian, A.; Morales, J. M.; Lu, A.; Mueller, J.; Davidson, Z. S.; Boley, J. W.; Lewis, J. A. *Advanced Materials* **2021**, *33* (27), 2101814.

[2] Bird, Robert Byron, Robert Calvin Armstrong, and Ole Hassager. "Dynamics of polymeric liquids. Vol. 1: Fluid mechanics." (1987), pp. 181-183.

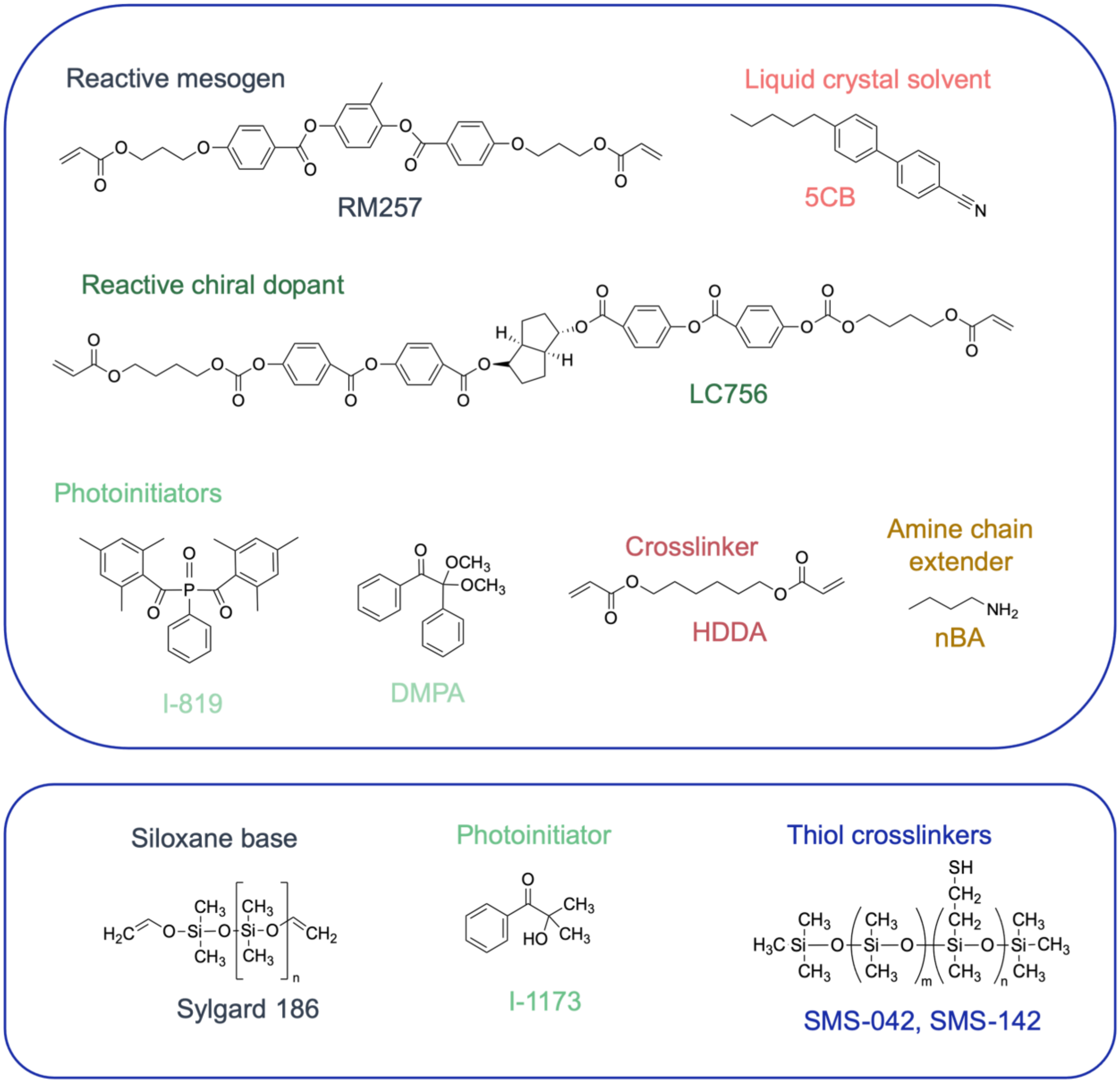

Figure S1. Chemical structures of components in CLC (top) and silicone (bottom) inks.

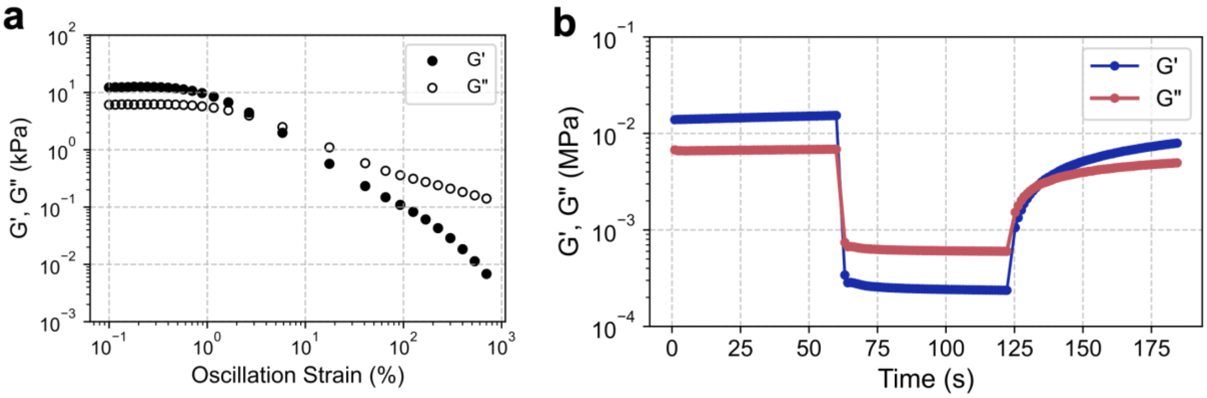

Figure S2. a) Log-log plot of the storage modulus, G’, and loss modulus, G”, as a function of oscillation strain for the silicone ink. b) Three-step oscillation method of G’ and G” of silicone ink at a 0.1% strain for 60 s, 30% strain for 60 s, followed by 0.1% strain for 70 s.

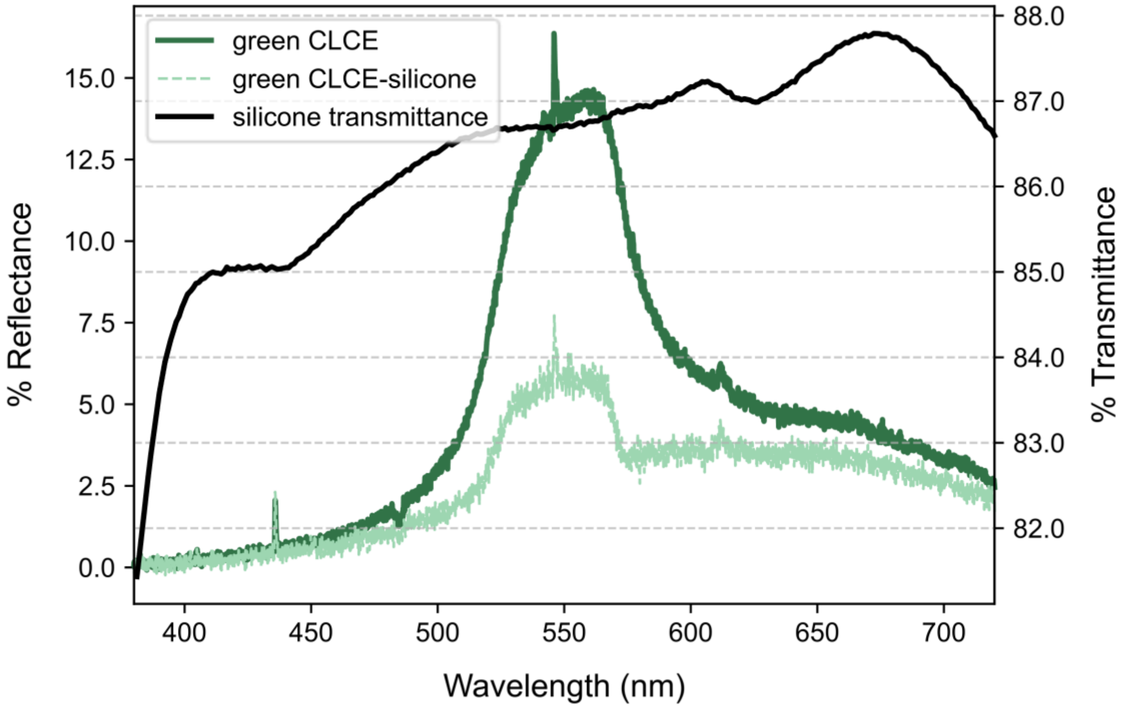

Figure S3. Reflectance (left) and transmittance (right) of a printed green CLCE bar and a printed green CLCE-silicone bar.

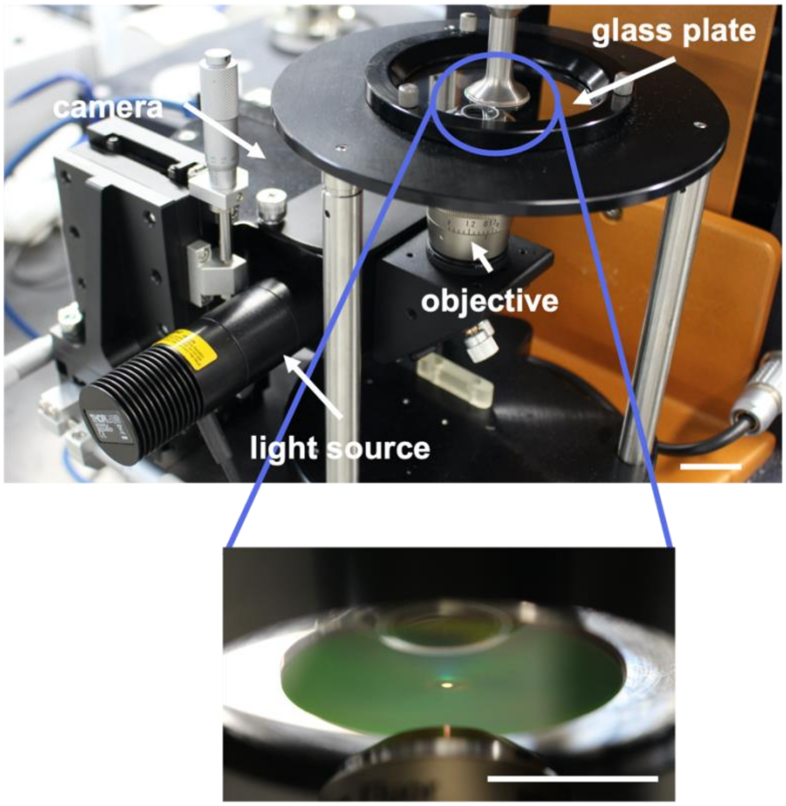

Figure S4. Image of the optical rheology setup. Scale bar, 1 cm.

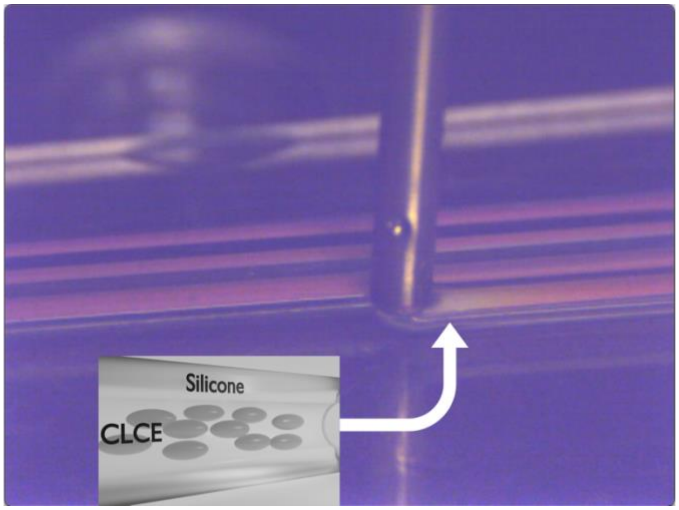

Figure S5. Snapshot image of the CLCE-silicone filament during coaxial printing along a snake pattern toolpath. Inset: Illustration of the pseudo-nematic phase present upon extrusion from the nozzle.

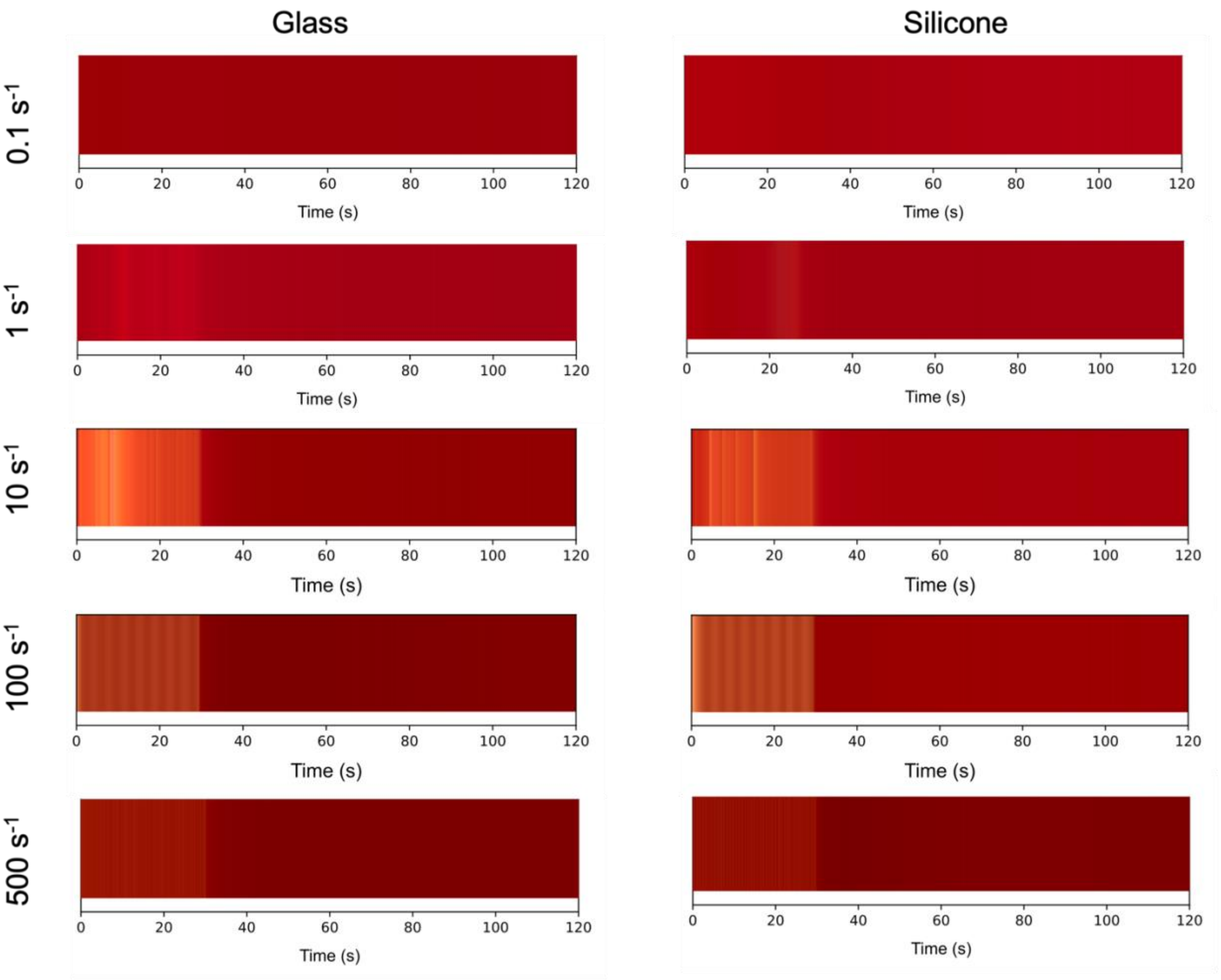

Figure S6. RGB values from the POM rheology videos are used to color vertical lines and form visuals representing the ink colors as a function of time and CLC shear rate.

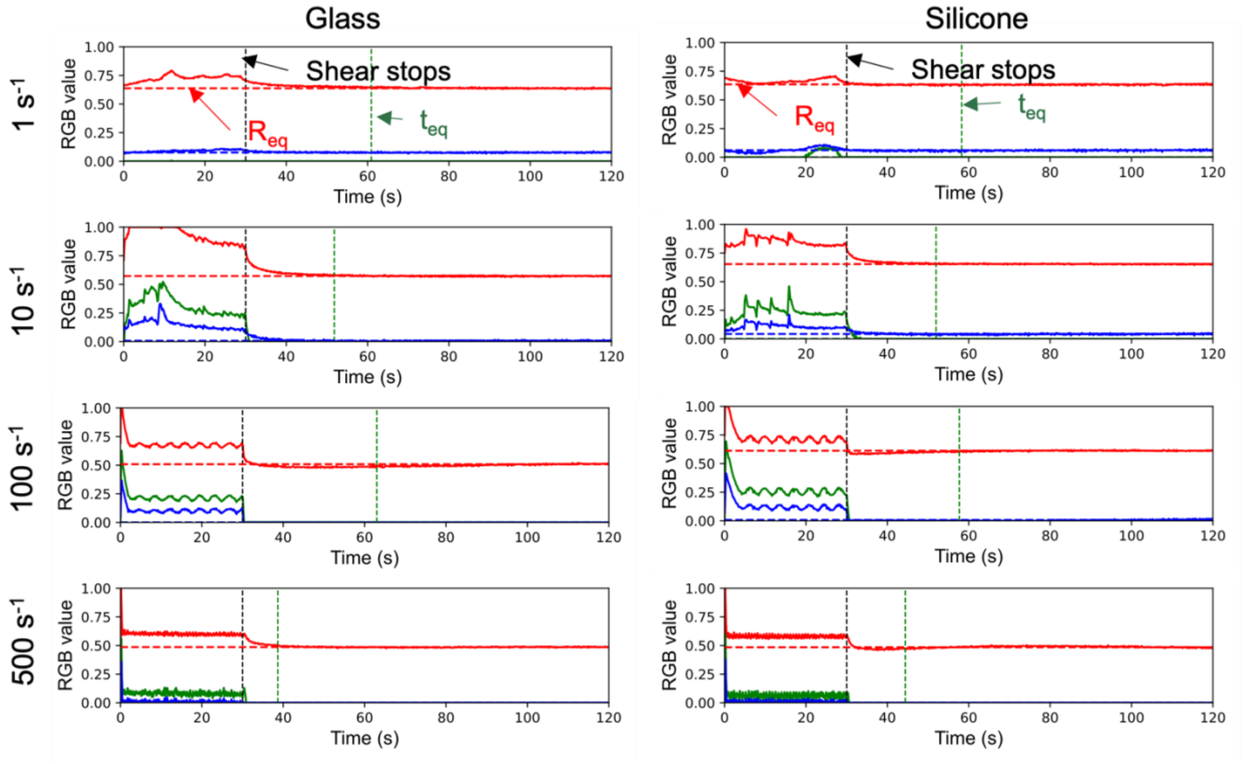

Figure S7. RGB values extracted from each frame of the POM rheology videos and plotted as a function of time and CLC shear rate.

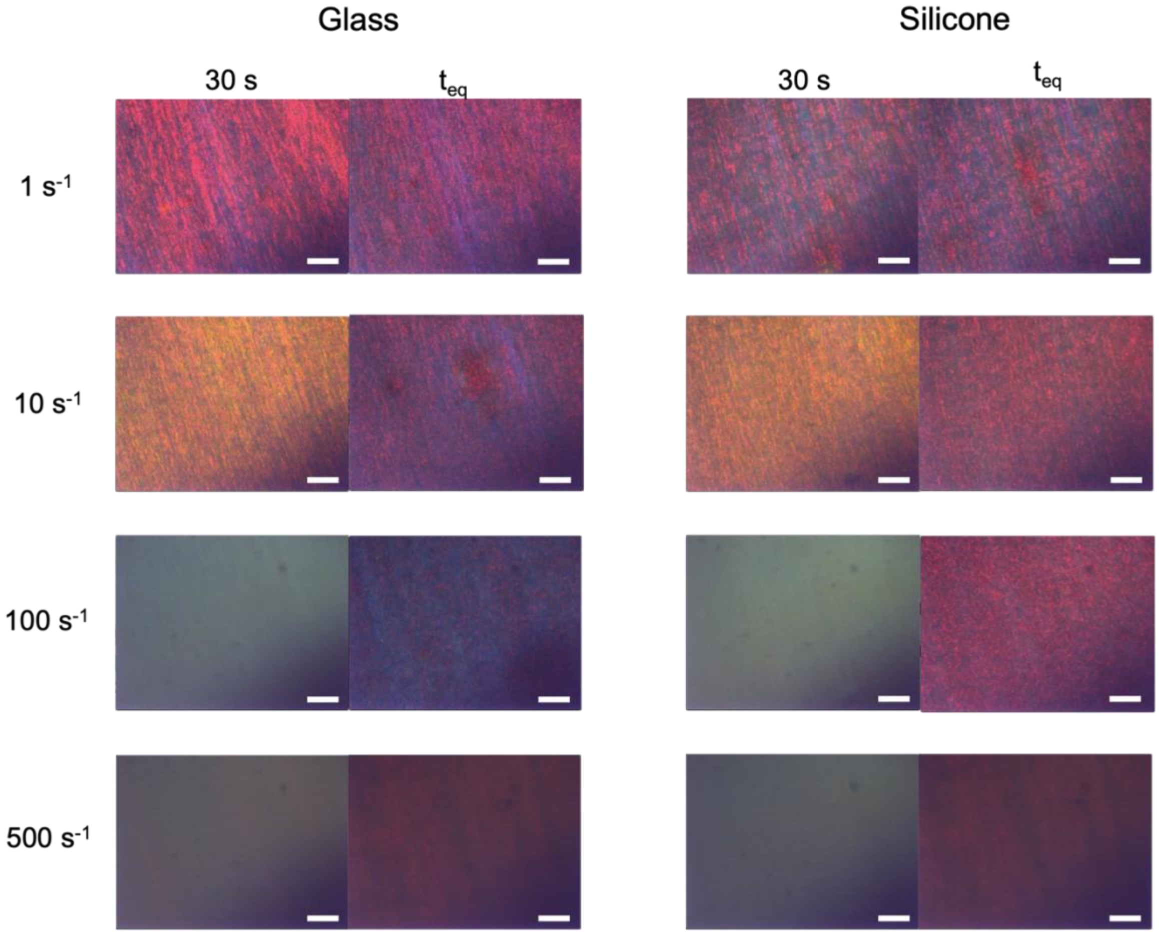

Figure S8. In-situ POM images showing the shear flow rheology of a red CLC ink sheared on top of a glass slide and a silicone film at varying shear rates. The images are taken after 30 s of shear and at the equilibrium time, t_{eq}. Scale bars, 100 µm.

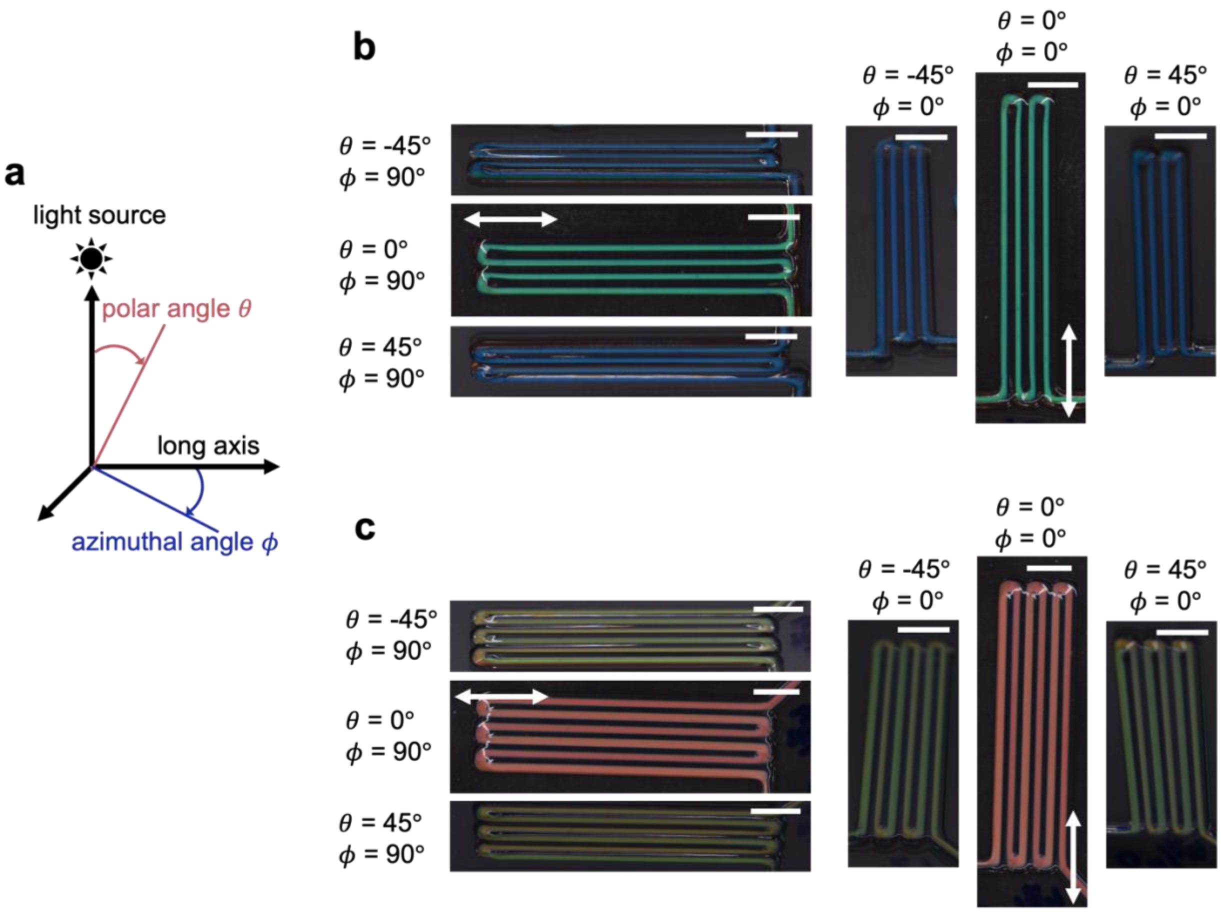

Figure S9. a) Drawing indicating the definition of the two angles θ and ϕ in relation to the long axis of the printed bars. b) Images of a green CLCE-silicone bar as seen from θ = -45°, 0°, or 45°, and ϕ = 0° or 90°. c) Images of a red CLCE-silicone printed bar as seen from θ = -45°, 0°, or 45°, and ϕ = 0° or 90°. The print direction is indicated with a double-pointed arrow. Scale bars, 5 mm.

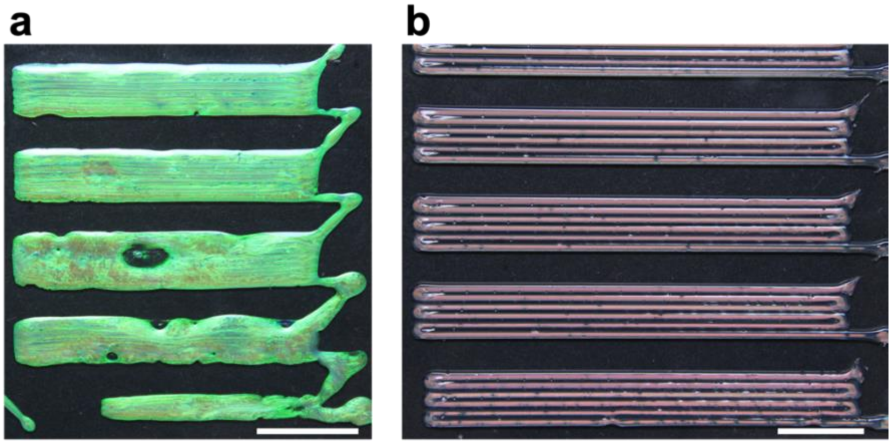

Figure S10. a) Printed green CLCE bars with air bubbles. b) Printed red CLCE-silicone bars with air bubbles. Scale bars, 1 cm.

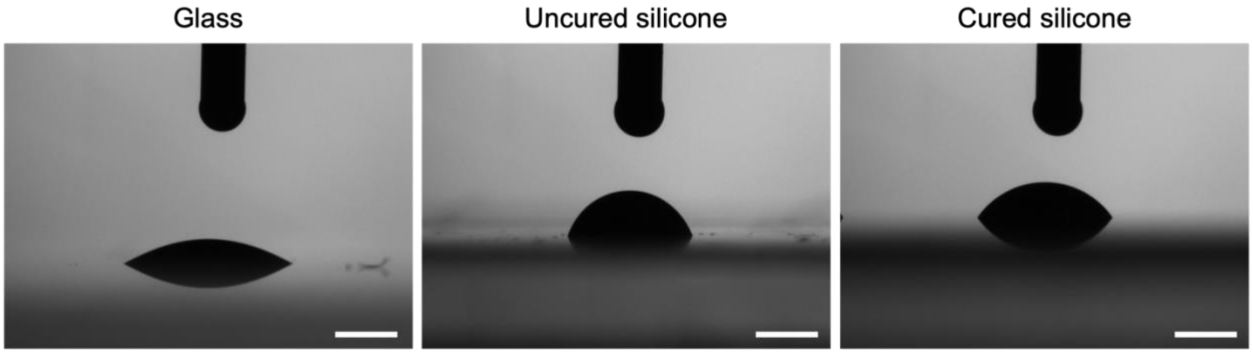

Figure S11. Optical images of the droplets of CLC ink on glass, uncured silicone, and silicone substrates, respectively. Scale bars, 1 mm.

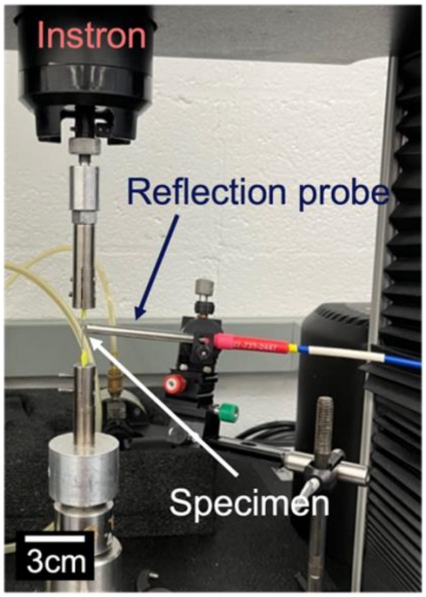

Figure S12. Photo of the Instron setup for tensile testing and spectroscopy characterization.

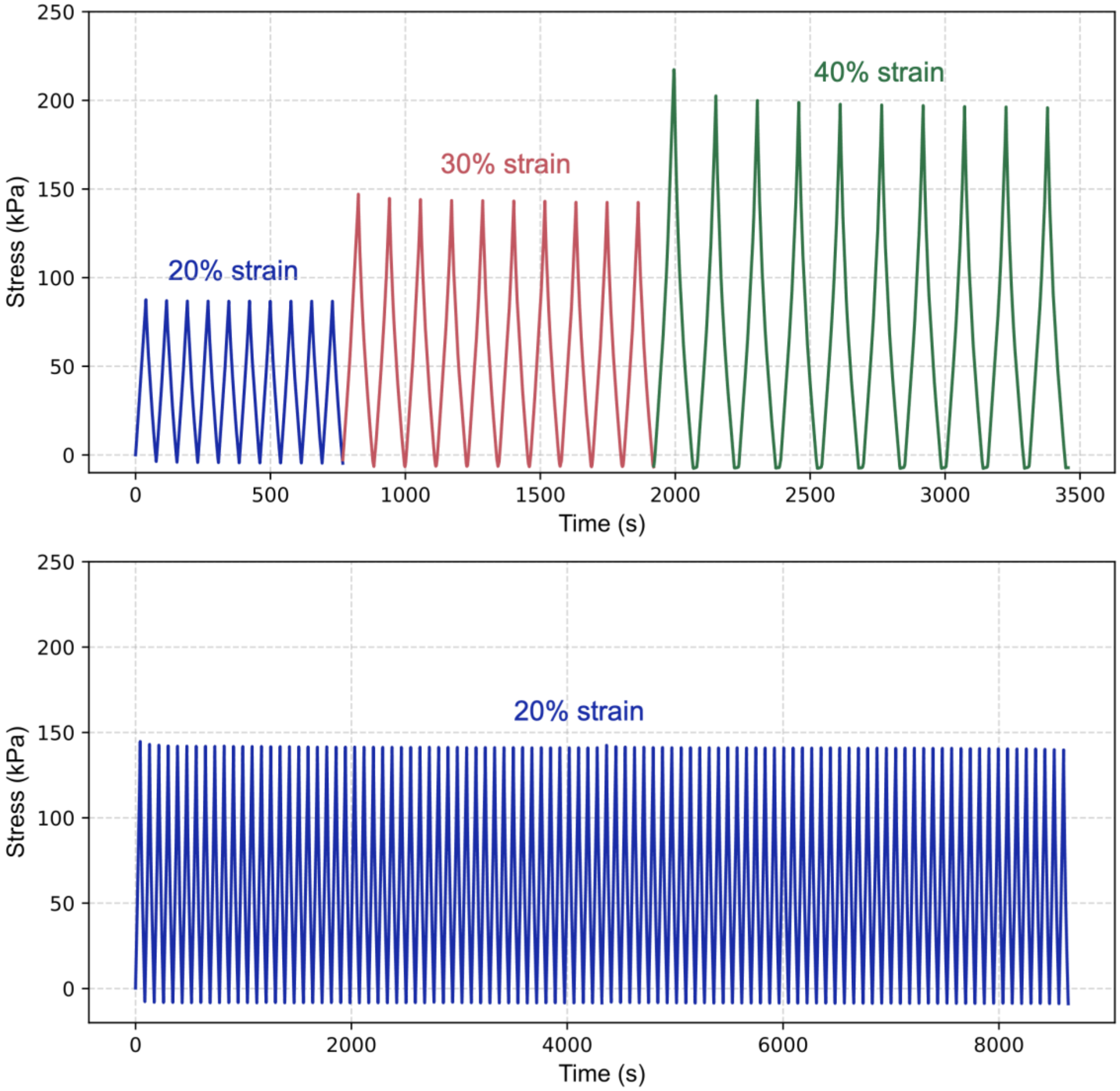

Figure S13. Cyclic testing of a green CLCE-silicone bar undergoing 20, 30, and 40% strains for 10 cycles each (top) and 20% strain for 100 cycles (bottom).

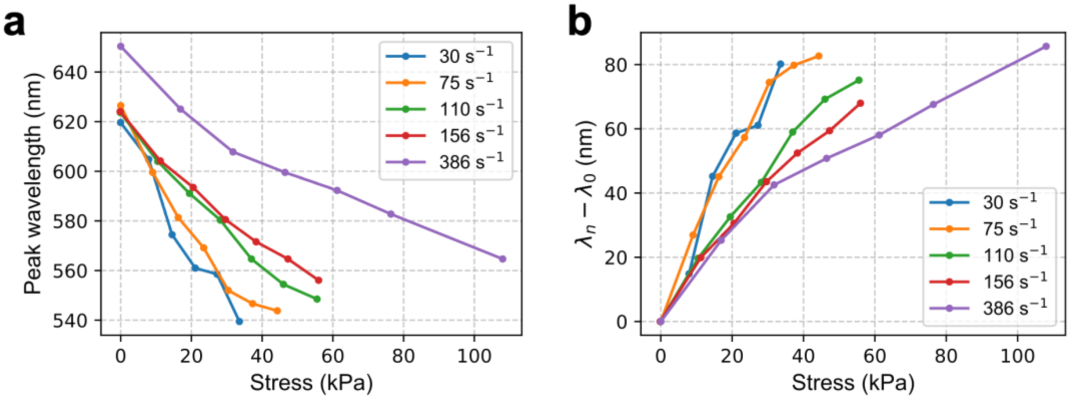

Figure S14. a) Peak wavelengths of the CLCE-silicone bars as a function of stress and CLC shear rates. b) Difference in peak wavelength between the applied stress and initial peak wavelength, $\lambda_n - \lambda_0$, for the red CLCE-silicone bars as a function of stress and CLC shear rates.

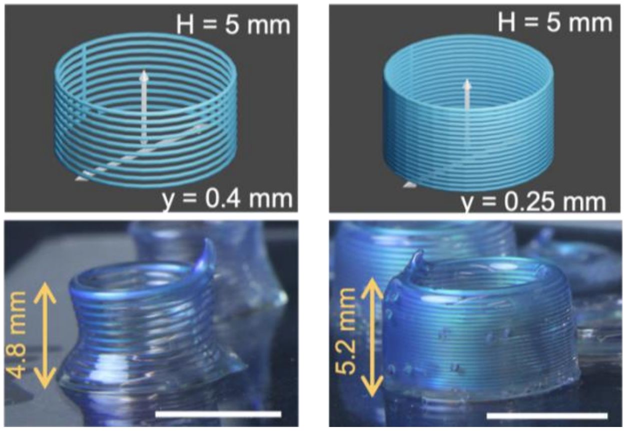

Figure S15. Optical images of CLCE-silicone bars printed with different layer heights (left: y = 0.4 mm, right: y = 0.25 mm). Corresponding print paths are shown above the images. Scale bars, 5 mm.

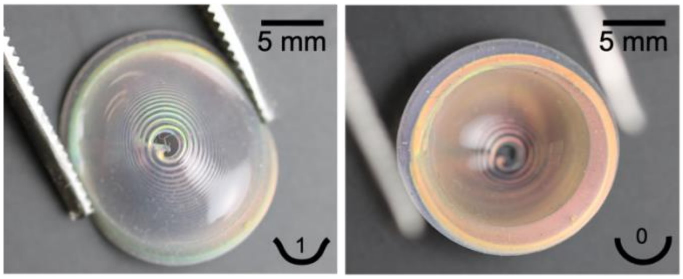

Figure S16. Optical images of the bottom face of a freeform CLCE-silicone dome. The numbers, 1 and 0, indicate whether the dome is inverted (held with tweezers) or not, respectively.

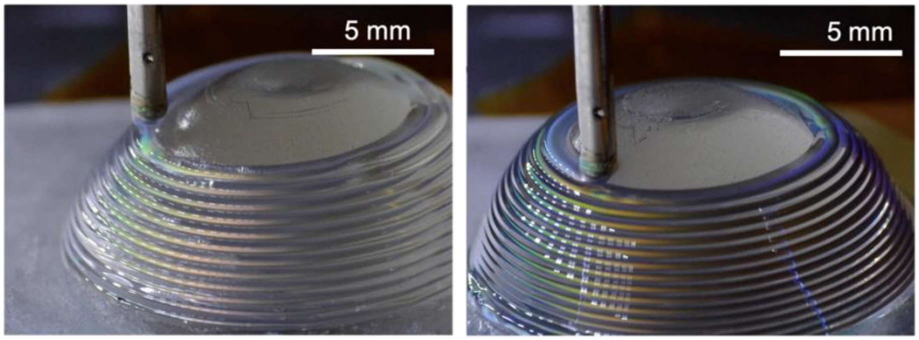

Figure S17. Optical images of CLCE-silicone being conformally printed while not centered properly (left) and centered properly (right).

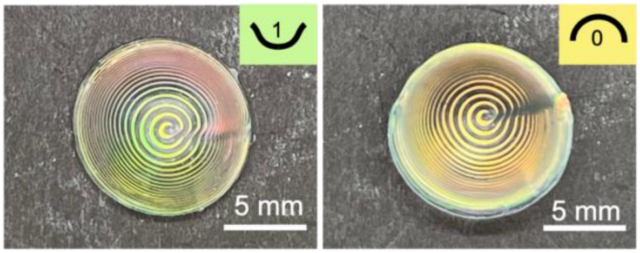

Figure S18. Optical images of the bottom face of a conformally printed CLCE-silicone dome. The numbers, 1 and 0, indicate whether the dome is inverted or not, respectively.

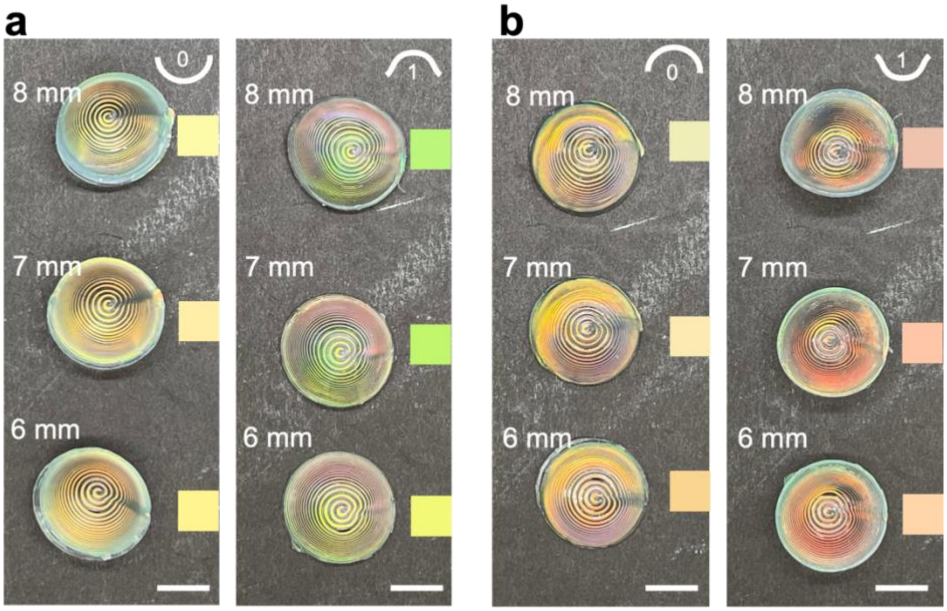

Figure S19. Optical images of the a) bottom face and b) top face of conformally printed CLCE-silicone domes with the substrate heights indicated. The numbers, 1 and 0, indicate whether the dome is inverted or not, respectively. The colors of the squares depict the corresponding colors of the centers of the faces. Scale bars, 5 mm.

Table S1. Dimensions of domes shown in Figure 6.

	Height (mm)	Radius (mm)
Dome 1 (6 mm)	7.40	9.15
Dome 2 (7 mm)	8.20	9.20
Dome 3 (8 mm)	9.15	9.40

*: indicating the height of the dome substrate.

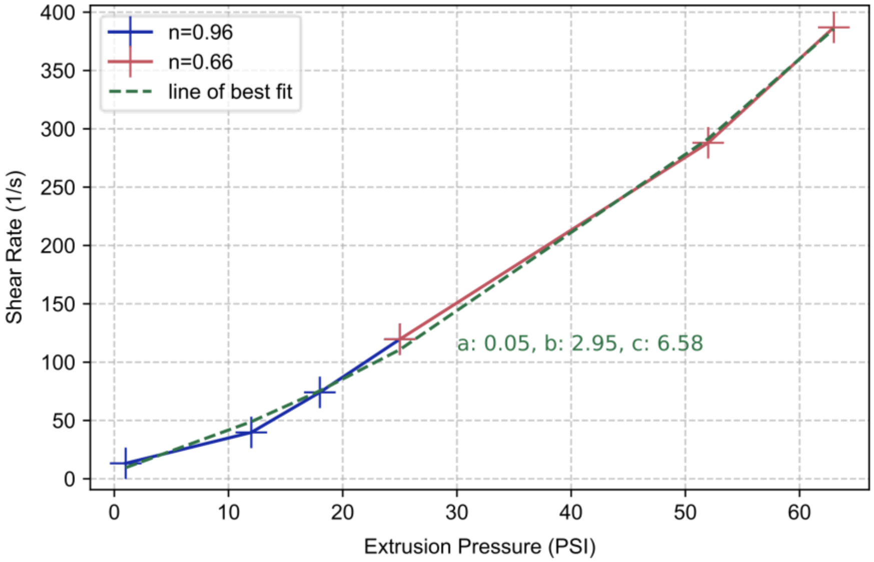

Figure S20. Wall shear rate for the core CLC ink as a function of extrusion pressure. The power law index, n, is calculated from the viscosity data. The line of best fit is shown in green, with coefficients a, b, and c indicated.

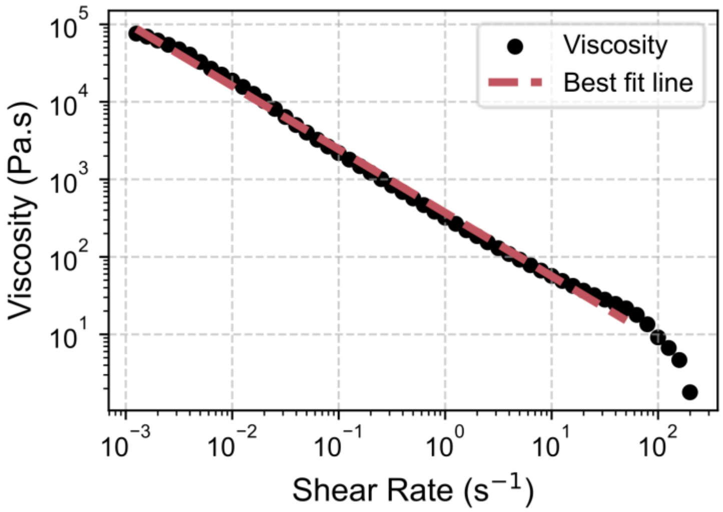

Figure S21. Viscosity of silicone ink as a function of shear rate. The power law index, *n*, is calculated from the line of best fit.

Movie S1. Shear test performed on a red CLC ink at 1s^{-1}, 10 s^{-1}, 100 s^{-1}, and 500 s^{-1} on a glass plate.
Movie S2. Shear test performed on a red CLC ink at 1s^{-1}, 10 s^{-1}, 100 s^{-1}, and 500 s^{-1} on a silicone-coated glass plate.
Movie S3. Cyclic testing of a green CLCE-silicone bar.
Movie S4. Inversion and snap-through of a freeform CLCE-silicone dome.
Movie S5. Inversion of conformally-printed CLCE-silicone domes.
Movie S6. Stretching of a three-dome mechanochromic strain sensor as seen from the top and side.
